# The Anti-Cancer Properties of the HIV Protease Inhibitor Nelfinavir

**DOI:** 10.3390/cancers12113437

**Published:** 2020-11-19

**Authors:** Mahbuba R. Subeha, Carlos M. Telleria

**Affiliations:** Experimental Pathology Unit, Department of Pathology, McGill University, Montreal, QC H3A 2B4, Canada; mahbuba.subeha@mail.mcgill.ca

**Keywords:** nelfinavir, cancer, endoplasmic reticulum stress, proteasome, cell cycle, apoptosis, Akt phosphorylation, autophagy, chemotherapy, drug repositioning

## Abstract

**Simple Summary:**

To this day, cancer remains a medical challenge despite the development of cutting-edge diagnostic methods and therapeutics. Thus, there is a continual demand for improved therapeutic options for managing cancer patients. However, novel drug development requires decade-long time commitment and financial investments. Repurposing approved and market-available drugs for cancer therapy is a way to reduce cost and the timeframe for developing new therapies. Nelfinavir is an anti-infective agent that has extensively been used to treat acquired immunodeficiency syndrome (AIDS) in adult and pediatric patients. In addition to its anti-infective properties, nelfinavir has demonstrated potent off-target anti-cancer effects, suggesting that it could be a suitable candidate for drug repurposing for cancer. In this review, we systematically compiled the therapeutic benefits of nelfinavir against cancer as a single drug or in combination with chemoradiotherapy, and outlined the possible underlying mechanistic pathways contributing to the anti-cancer effects.

**Abstract:**

Traditional cancer treatments may lose efficacy following the emergence of novel mutations or the development of chemoradiotherapy resistance. Late diagnosis, high-cost of treatment, and the requirement of highly efficient infrastructure to dispense cancer therapies hinder the availability of adequate treatment in low-income and resource-limited settings. Repositioning approved drugs as cancer therapeutics may reduce the cost and timeline for novel drug development and expedite the availability of newer, efficacious options for patients in need. Nelfinavir is a human immunodeficiency virus (HIV) protease inhibitor that has been approved and is extensively used as an anti-infective agent to treat acquired immunodeficiency syndrome (AIDS). Yet nelfinavir has also shown anti-cancer effects in in vitro and in vivo studies. The anti-cancer mechanism of nelfinavir includes modulation of different cellular conditions, such as unfolded protein response, cell cycle, apoptosis, autophagy, the proteasome pathway, oxidative stress, the tumor microenvironment, and multidrug efflux pumps. Multiple clinical trials indicated tolerable and reversible toxicities during nelfinavir treatment in cancer patients, either as a monotherapy or in combination with chemo- or radiotherapy. Since orally available nelfinavir has been a safe drug of choice for both adult and pediatric HIV-infected patients for over two decades, exploiting its anti-cancer off-target effects will enable fast-tracking this newer option into the existing repertoire of cancer chemotherapeutics.

## 1. Introduction

Human immunodeficiency virus (HIV) protease inhibitors (PIs) are a group of drugs designed to target the aspartyl protease enzyme of the virus. The ribonucleic acid (RNA) in HIV encodes for two polyproteins—gag and gag-pol—which are cleaved at specific regions by an aspartyl protease for the maturation of the nascent virions through morphologic changes and condensation of the nucleoprotein core [1]. To date, ten HIV-PIs have been approved by the United States Food and Drug Administration (FDA); they contain a synthetic analogue of the gag-pol polyprotein, having a sequence of phenylalanine-proline at 167 and 168 regions [2,3]. The HIV-PIs currently available in the market are nelfinavir, saquinavir, ritonavir, indinavir, amprenavir, fosamprenavir, lopinavir, atazanavir, darunavir, and tipranavir [3,4]. The HIV-PIs exert their therapeutic benefit by inhibiting subsequent HIV infection in a patient; however, they do not exert any action on cells already carrying integrated proviral DNA [1]. Thus, HIV-PIs have been in use in combination with reverse transcriptase inhibitors to treat HIV-infected patients, constituting the standard protocol of highly active antiretroviral treatment (HAART) [5].

Rational drug design of the HIV-PIs as peptidomimetics—based on the amino acid sequence recognized by the HIV aspartyl protease—was intended to drive competitive binding of the drug at the active site of the enzyme and disrupt the enzyme–substrate reaction [6]. Mammalian aspartyl proteases are weaker in cleaving and inhibiting maturation of HIV polyproteins than the HIV-residing enzyme; thus, it was expected that the HIV-PIs would spare the human proteases and induce minimal toxicity. However, soon after the introduction of the HIV-PIs in the HAART protocol, pleiotropic off-target effects of the HIV-PIs were reported. The emergence of reports of remission from AIDS-associated cancers suggested anti-neoplastic properties of HIV-PIs to be a potentially important off-target effect. For instance, Niehuse et al. reported a case of complete regression of AIDS-associated Kaposi’s sarcoma (KS) in a 5-year-old child undergoing HAART regimen consisting of HIV-PI nelfinavir and reverse transcriptase inhibitors zidovudine and lamivudine [7]. Lebbé [8] and Krischer [9] also reported regression of KS in HIV-infected adults undergoing combination therapies of HIV-PIs and reverse transcriptase inhibitors. Initially, the reduction in AIDS-associated cancers was attributed to the immune reconstitution of the body as a result of improved CD4+ T cell count and the reduction in overall viral load; however, later reports suggested that direct off-target anti-cancer action by HIV-PIs could be possible. Sgadari et al. suggested that the antiangiogenic properties of indinavir and saquinavir contributed to the regression of Kaposi’s sarcoma in mice models [10,11], whereas Schmidtke et al. demonstrated that ritonavir could affect the cellular proteasome activity in addition to its immunomodulatory and virus-reducing actions [12]. Thus, multiple preclinical reports suggesting the pleotropic effects of HIV-PIs initiated the research for their possible anti-neoplastic properties.

Nelfinavir is a first-generation HIV-PI, which was approved by the FDA in March 1997 [13,14] for treating HIV infection. Due to the emergence of second- and third-generation HIV-PIs, nelfinavir has been progressively displaced from the HAART protocol [15]; however, nelfinavir exhibited maximum anti-neoplastic efficiency among the HIV-PIs. Wu et al. suggested that a unique *cis*-decahydroisoquinoline-2 carboxamide moiety may be responsible for the higher anti-neoplastic efficiency of nelfinavir. Analysis through a bioinformatical virtual docking system suggested that nelfinavir can potentially bind at the ATP binding site of the EGFR (ERBB1) protein, which was structurally compared with the same-site binding of the EGFR inhibitor lapatinib [16]. Further molecular docking approaches predicted the probability of binding of nelfinavir with cellular kinases [17] and Hsp90β protein [18], which may also contribute to its anti-cancer properties. In 2007, in a landmark paper by Gills et al., the preclinical anti-neoplastic efficiency of nelfinavir was demonstrated in the NCI60 cancer cell panel [19].

Long-term treatment with nelfinavir in HIV-infected patients led to adverse events such as hyperglycemia, insulin resistance, and lipodystrophy, denoting mechanisms of action of nelfinavir disparate from its anti-viral activity [1]. One of the mechanisms by which insulin resistance is triggered in the body is by the inhibition of the IGF/Akt pathway, which is upregulated in many cancers. Thus, from the observation of insulin resistance, it was postulated that nelfinavir could act as an inhibitor of the Akt pathway in cancer, which was later demonstrated in preclinical studies [19]. To date, multiple research groups have used multipronged approaches to understand and implement the anti-cancer properties of nelfinavir in preclinical settings and clinical trials, with the aim of repositioning the drug as a potential chemotherapeutic agent against a multitude of cancers.

Repositioning already approved drugs for cancer therapeutics is desirable for two reasons: to reduce the timeframe of the drug development pipeline, and to increase the affordability of chemotherapeutics for patients. At present, it takes approximately a decade to go from target identification to FDA approval of a new drug, and these new drugs themselves remain cost prohibitive for large segments of the population, especially in low-income countries. Data available from preclinical studies and toxicity profiling may contribute to the rapid repurposing of nelfinavir in the clinical setting. Furthermore, the recent emergence of nelfinavir in generic form [20] following patent expiration may reduce the cost of treatment as a result of drug repurposing. Minimal toxicity in clinical trials and ease of introduction through oral route may also be an important consideration for repurposing nelfinavir.

This review offers a systematic analysis of the studies investigating the role and efficiency of nelfinavir against a plethora of cancers in preclinical settings and clinical trials.

## 2. Potential Mechanisms Whereby Nelfinavir Exert Its Anti-Cancer Effect

### 2.1. Cell Cycle Arrest

Nelfinavir has been shown to inhibit cellular proliferation in multiple cancers, and a number of studies focused on the ability of nelfinavir to regulate the cell cycle. Bruning et al. reported that nelfinavir reduced the level of cell cycle proteins cyclin A, cyclin B, cyclin D3, cyclin-dependent kinase (CDK) 1, CDK2, and proliferating cell nuclear antigen (PCNA) in ovarian cancer cell lines in a time-dependent manner [21]. The authors further reported nelfinavir-mediated reduction in cyclin B and CDK1 in leukemia cells, which was associated with a reduction in cells in the G2/M phase and a striking increase in cells with sub-G1 DNA content, suggesting an effect of nelfinavir on both the apoptotic pathway and the cell cycle [22]. A similar result was observed in cervical cancer cells, where nelfinavir-treated cells showed a decrease in S phase with a marked increase in sub-G1 DNA content. The changes were accompanied by decreased expression of cyclins D3 and B in nelfinavir-treated cells. The authors further observed an increase in the cell-cycle regulatory and pro-apoptotic protein p53 in nelfinavir-treated cervical cancer cells carrying the wild-type p53 gene [23]. Chow et al. demonstrated that nelfinavir caused accumulation of liposarcoma and fibrosarcoma cells in the G1 phase of the cell cycle, which was associated with increased expression of cell cycle inhibitor p21, and decreased level of PCNA [24]. Jiang and colleagues [25] reported a significant accumulation of nelfinavir-treated melanoma cells also in the G1 phase; a dose of 15 μM nelfinavir caused a time-dependent decrease in the kinase activity of CDK2 in the melanoma cells, which was attributed to the reduced activity of CDK2-specific phosphatase Cdc25A, because removal of the inhibitory phosphate groups at the Thr^14^ and Thr^15^ positions by Cdc25A renders CDK2 fully active. These authors suggested that proteasome-mediated degradation of Cdc25A was responsible for the reduced activity of CDK2, resulting in the G1 arrest of the melanoma cells. A reduced CDK2 activity resulted in reduced phosphorylation of the Rb protein at the Ser^608^ position. Reduced phosphorylation of Rb inhibits its dissociation from the transcription factor E2F, making it impossible for the cells to cross the restriction point and enter the S phase [25]. Jensen et al. reported G1 arrest of thyroid cancer cells in response to nelfinavir in a dose-dependent manner with concomitant reduction in the level of CDK4, cyclin D1, and phospho-Rb [26]. Sato and colleagues reported dose-dependent reduction in cyclin D1 and CDK4 in bladder cancer cells in response to nelfinavir monotherapy. A robust increase in sub-G1 DNA content was observed during combination therapy with nelfinavir and ritonavir in such cells [27]. In similar experiments, Okubo et al. described nelfinavir-mediated dose-dependent accumulation of sub-G1 DNA content in renal cancer cells with concomitant reduction in cyclin D1 and CDK4—a phenomenon further aggravated by the addition of panobinostat, an inhibitor of histone deacetylases (HDAC) [28,29]. Soprano et al. reported slight accumulation of breast cancer cells in the G1 phase following treatment with nelfinavir for 24 h, associated with a clear reduction in cell cycle regulatory proteins cyclin D, E, A, B and phospho-Rb, and with an increase in the cell cycle inhibitory protein p21; strikingly, the cell cycle regulatory effects of nelfinavir observed in breast cancer cell lines were not evident in healthy breast epithelial cells [30]. In hepatocellular carcinoma cells (HCC), a 24 h treatment with varying doses of nelfinavir resulted in G1 arrest; however, the changes in underlying regulatory proteins were not explored [31]. Veschi et al. observed nelfinavir-mediated G1 arrest of pancreatic cancer cells in a cell type-specific manner; protein levels of cyclin D3 and B1 were downregulated in response to nelfinavir monotherapy in pancreatic cancer cells, and were further decreased when nitroxoline and erlotinib were added to the treatment [32]. Xiang and colleagues observed a dose-dependent G1 arrest of cervical cancer cells in response to nelfinavir with a concomitant dose-dependent reduction in cell proliferation observed through a BrdU incorporation assay. The authors suggested a role of oxidative stress in cell cycle regulation following nelfinavir treatment, as they observed a reversal of the inhibition of nelfinavir-mediated cell proliferation during co-treatment with the reactive oxygen species (ROS) scavenger N-acetylcysteine (NAC) [33]. It was also reported that cervical cancer cells accumulate in the G2/M phase following co-treatment with nelfinavir and metformin, which was associated with increased expression of p53 and p21 [34]. Taken together, the reports indicate that the effects of nelfinavir on the cell cycle may be specific to the cancer cell type, and, in most instances, is an early event during treatment, which precedes the induction of cell death pathways.

### 2.2. Cell Death

Nelfinavir-induced cell death in cancer cells is evident in many studies; however, the death modalities seem to be different depending on the cancer cell types and the experimental conditions used. Flow cytometric analysis of nelfinavir-treated lung cancer cells H157 and A549 revealed that nelfinavir increased the percentage of sub-G1 DNA contents more potently than in cells treated with ritonavir and saquinavir, indicating a superior anti-cancer potency of nelfinavir compared to other HIV-PIs. Increased sub-G1 DNA contents and pyknotic nuclei in the nelfinavir-treated lung cancer cells were associated with the cleavage of caspase-8 and caspase-9, suggesting the activation of both extrinsic and intrinsic apoptotic pathways. At the downstream level, the activation of caspase 9 and 8 converged into the cleavage of executioner caspases—caspase-3 or caspase-7 or both, which further cleaved the apoptotic target poly ADP-ribose polymerase (PARP) [19,35]. To determine whether caspase activation is imperative to cell death induced by nelfinavir treatment on cancer cells, a pan-caspase inhibitor, zVAD, was applied during treatment with nelfinavir on lung cancer cells; zVAD reduced nelfinavir-induced sub-G1 DNA content, at least in part confirming a nelfinavir-induced caspase-dependent cell death mechanism.

Cell death induced by nelfinavir in lung cancer cells was also associated with the induction of endoplasmic reticulum (ER) stress and autophagy, while the inhibition of autophagy by 3-methyladenine (3MA) further increased the number of dead cells, suggesting a compensatory protective role of autophagy [19,35]. It is possible that a shift in the balance of the pro-death and pro-survival mechanisms during nelfinavir treatment commands the ultimate fate of the cancer cells, which could explain the parallel activation of autophagy during nelfinavir-induced cell death [19]. Collateral activation of cell-protective mechanisms during impending death has been also reported in nelfinavir-treated ovarian and leukemia cells. The authors demonstrated the upregulation and increased phosphorylation of mitochondrial protective anti-apoptotic protein mcl-1 in cancer cells in response to nelfinavir, which was decreased during co-treatment with sorafenib—a known downregulator of mcl-1, contributing to further reduction in cell survival [22,35]. Mitochondrial membrane potential was unaltered in both ovarian cancer and leukemia cells during nelfinavir treatment; however, activation of caspases 8, 9, 7, and 3 and the cleavage of downstream PARP were evident in leukemia cells [22,35]. Contrary to the reports of Bruning et al. [22,35], Xiang and colleagues observed a reduction in the mitochondrial membrane potential during nelfinavir inflicted death on cervical cancer cells [33]. The increased number of apoptotic cervical cancer cells treated with nelfinavir was associated with an increased production of ROS, which predominantly originated from the membrane-compromised mitochondria. The addition of a mitochondria-targeted antioxidant reduced the number of apoptotic cervical cancer cells treated with nelfinavir, indicating an important role of mitochondrial ROS in nelfinavir-induced cell death.

Immunoblots revealed the localization of apoptosis-inducing factor (AIF)—a pro-apoptotic mitochondrial flavoprotein—in the nucleus and the reduction in its level in the mitochondrial extracts of cervical cancer cells treated with nelfinavir [33]. Translocation of AIF from the mitochondria to the nucleus has been implicated in caspase-independent cell death [36]. Xiang et al. concluded that nelfinavir was able to induce apoptosis in a caspase-independent manner through ROS production and AIF translocation. Additionally, the nelfinavir-mediated apoptosis in this case was not abolished when the pan-caspase inhibitor zVAD was added, which further proved the concept of caspase-independent cell death [33]. Soprano and colleagues also observed a concomitant rise in ROS production during nelfinavir-induced death in breast cancer cells. In response to nelfinavir, the cells had an increased level of pro-apoptotic Bak protein and a reduction in the level of procaspase-9, which was associated with an increased level of mitochondrial cytochrome c in cytosolic lysates, indicating the activation of the intrinsic apoptotic pathway [30].

Activation of classical apoptotic pathways following nelfinavir treatment has been reported in a number of studies. Cleavage of caspase-3 has been reported after nelfinavir monotherapy in multiple myeloma (MM) and thyroid cancer cells [26,37,38]. Bruning et al. described apoptosis in both estrogen receptor-positive and -negative breast cancer cells associated with PARP cleavage during nelfinavir therapy [39], which was also evident in chemotherapy sensitive and resistant breast cancer cells [40]. The combination of nelfinavir and dimethylcelecoxib (DMC)—a close structural analog of celecoxib that lacks cyclooxigenase-2 (COX2) inhibitory function—resulted in enhanced cleavage of caspase-7 and PARP in breast cancer cells [40]. During triple therapy with nelfinavir, DMC, and chloroquine, Thomas et al. observed a reduction at colony formation in triple-negative breast cancer (TNBC) cells, which was associated with the cleavage of caspases 3 and 7. The authors further observed an increase in apoptotic cells in tumors derived from TNBC xenografts identified by the positive terminal deoxynucleotidyl transferase (TdT) dUTP Nick-End Labelling (TUNEL) assay [41]. Davis and colleagues reported cleavage of caspase-7 in nelfinavir-treated cisplatin-sensitive and -resistant cervical cancer cells, which corroborated similar findings in breast cancer cells [42,43]. In pediatric leukemia cells treated with nelfinavir, PARP cleavage was associated with the cleavage of upstream apoptosis initiator caspase-9 [44]. Liu et al. observed a resensitization of doxorubicin-resistant chronic myeloid leukemia (CML) cells during co-treatment of suboptimal doses of nelfinavir with doxorubicin, which resulted in increased apoptosis associated to caspase-3 cleavage, increased proapoptotic protein Bax, and decreased anti-apoptotic protein Bcl-2 [45]. In castration-resistant prostate cancer cells, nelfinavir did not activate caspase-3 at low doses; however, in combination with docetaxel and curcumin, caspase-3 was activated, which resulted in DNA fragmentation and cleavage of PARP [46]. Positive TUNEL cells were enhanced in tumors derived from castration-resistant prostate cancer xenografted mice treated with nelfinavir, curcumin, and docetaxel, compared to untreated controls [46]. Yang et al. also observed potentiation of toxicity among nelfinavir and docetaxel in non-small-cell lung carcinoma (NSCLC) cells, which was associated with increased TUNEL-positive cells and a reduction in the anti-apoptotic protein Bcl-2 [47]. Increased TUNEL-positive cells were also observed during nelfinavir treatment in prostate cancer cells in vitro and in vivo [48]. In HCC, dual treatment of nelfinavir and proteasome inhibitor oprozomib resulted in enhanced activation of caspase 3/7 compared to individual therapy with oprozomib. Increased TUNEL-positive cells were present in the diethylnitrosamine (DEN)-induced hepatotoxic model of HCC xenografted mice having received nelfinavir and oprozomib treatment, compared to the control group [49]. Nelfinavir, alone and in conjunction with nitroxoline (antibiotic with anti-cancer properties) and erlotinib (EGFR inhibitor), resulted in reduced cell viability, PARP cleavage, and colony formation in pancreatic cancer cells [32]. Gupta and colleagues demonstrated that nelfinavir reduced the level of pro-survival protein survivin and increased proapoptotic protein Bax in meningioma cells, and that the effects were synergistically aggravated in combination with tyrosine kinase inhibitor imatinib. In vivo, tumors from meningioma xenografts showed increased TUNEL-positive cells in groups receiving dual treatment of nelfinavir and imatinib [50]. In renal cancer cells, Okubo et al. observed that nelfinavir-induced cell death associated with PARP cleavage, enhanced protein level of pro-apoptotic NOXA, and a gradual decrease in pro-survival protein survivin [28]. A high dose of nelfinavir further potentiated renal cancer cell death by the HDAC inhibitor panobinostat [29].

Activation of death receptor-mediated extrinsic apoptotic pathways has been implicated during nelfinavir therapy on multiple cancer types. The transmembrane death receptors belong to the tumor necrosis factor gene (TNF) superfamily. Among different ligands, tumor necrosis factor-related apoptosis-inducing ligand (TRAIL) has been characterized to induce death upon binding with corresponding death receptors (DR)—DR4/TRAIL-R1 and DR5/TRAIL-R2 [51]. Receptor–ligand interaction leads to downstream recruitment of an adaptor protein—Fas-associated protein with death domain (FADD)—and promotes subsequent recruitment and activation of initiator caspase-8. Aggregation and activation of caspase-8 culminate with the activation of executioner caspases to drive apoptosis. TRAIL has been considered an important addition to the anti-cancer drug inventory, and recombinant human TRAIL and monoclonal antibodies targeting TRAIL receptors have been promoted as chemotherapeutics [51,52]. Nelfinavir has been shown to enhance the expression of DR5 receptors in p53 mutant glioblastoma cells; however, it was not sufficient to induce death as a monotherapy. However, combination of nelfinavir and TRAIL promoted potent transactivation of DR5, which induced cell death in glioblastoma cells evidenced by increased sub-G1 DNA content, activation of caspases 8,9,3, and cleavage of PARP. The authors further demonstrated that nelfinavir-mediated potentiation of TRAIL was mediated by endoplasmic reticulum (ER) stress-related transcription factors ATF4 and CHOP [52]. DR5 is a downstream target of the p53 protein; thereby, p53 mutation may render resistance to TRAIL in cancer cells. However, the ability of nelfinavir to increase DR5 in a p53-independent manner can be used as a tool to increase TRAIL sensitivity in p53-mutated cancer cells [52]. Okubo et al. also demonstrated nelfinavir-mediated potentiation of TRAIL in renal cancer cells, where the decrease in viability during combination of TRAIL and nelfinavir was reversed by the addition of DR4 and DR5 blocking antibodies. The authors also demonstrated dose-dependent upregulation of both DR4 and DR5 receptors in response to nelfinavir in renal cancer cells [28]. Bruning et al. demonstrated that nelfinavir increased the mRNA level of DR5 in ovarian cancer cells within 48 h while the level of membrane resident DR5 increased after 48 to 72 h. Nelfinavir was also shown to potentiate the cytotoxic effects of TRAIL in ovarian cancer cells [53]. Similarly, nelfinavir-mediated upregulation of DR5 and sensitization to TRAIL was observed in cervical cancer cells [23]. Chow et al. observed an increased level of Fas—another death receptor which initiates extrinsic apoptosis upon binding with Fas ligand—and pro-apoptotic protein Bax in liposarcoma cells treated with nelfinavir [24].

### 2.3. Endoplasmic Reticulum (ER) Stress and Unfolded Protein Response (UPR)

ER stress is a cellular condition induced by an imbalance in cellular protein homeostasis. Internal and external noxious stimuli can lead to the accumulation of misfolded proteins in the ER lumen, which instigates an adaptive unfolded protein response (UPR) aiming at reducing the protein load, and restoring cellular homeostasis by correct refolding of proteins [54,55]. ER-resident chaperone of 78 kDa, glucose-regulated protein (GRP78) is responsible for detecting intraluminal misfolded proteins, leading to the activation of ER stress sensors inositol-requiring enzyme 1-α (IRE1α), protein kinase RNA-like endoplasmic reticulum kinase (PERK), and activating transcription factor 6 (ATF6), which are the upstream components of the UPR. At the downstream level, three outcomes can be expected initially: global inhibition of protein synthesis to reduce overall protein load, enhanced and selective synthesis of chaperone proteins to facilitate protein re-folding, and degradation of proteins mediated by the proteasome. Late-stage or exhaustive ER stress shifts from a pro-survival to a lethal mode initiating cell death [55]. ER stress has been frequently associated with cancer cells because glucose shortage and cellular hypoxia—two factors stimulating ER stress—are also known as important facilitators of tumor growth. Elevated ER stress in surviving cancer cells provides a therapeutic window for ER stress-stimulating chemotherapeutic drugs, as the drug-amplified ER stress can lethally target the cancer cells sparing the healthy cells, having no or minimal ER stress [55].

Nelfinavir has demonstrated potent ER stress-modulating effects against cancer cells in multiple studies. In time-dependent experiments, Gills et al. demonstrated phosphorylation of eukaryotic initiation factor 2α (eIF2α), a downstream effector of PERK, and enhanced expression of ER stress-related proteins such as transcription factor 3 (ATF3) and CCAAT enhancer-binding protein homologous protein (CHOP) in nelfinavir-treated lung, breast, and prostate cancer cells [19]. The authors also reported synergistic aggravation of ER stress markers in NSCLC and multiple myeloma (MM) cells during combined treatment of nelfinavir and the proteasome inhibitor bortezomib. It was implicated that ER stress played a crucial role in inducing cytotoxicity, since the silencing of ER stress-related proteins ATF3, CHOP, and PERK resulted in the reduction in cell death [56]. Bono et al. reported that nelfinavir, as a monotherapy, also increased the expression of CHOP and ATF4 in MM cells [37]. Further, in a NSCLC xenograft model, combined treatment of nelfinavir and bortezomib showed increased protein levels of ER stress markers GRP78, CHOP, p-eIF2α, and X-box binding protein-1 (XBP-1) [57]. In malignant glioblastoma cells, Pyrko et al. discovered a nelfinavir-mediated increase in the expression of GRP78, CHOP, and ER stress-related death mediator caspase-4; the importance of ER stress in nelfinavir-derived cytotoxicity was further underscored when siRNA-mediated silencing of GRP78 reduced the clonogenic survival [58]. Cho et al. also observed a dose-dependent increase in the ER stress-related proteins GRP78 and CHOP in breast cancer cells (MCF7, BT-474) and in their chemotherapy-resistant counterparts. The authors also observed that siRNA-mediated reduction in GRP78 contributed to reduced colony formation in nelfinavir-treated chemosensitive and -resistant breast cancer cells underpinning the ER stress-driven cytotoxicity of nelfinavir [40]. Furthermore, Bruning et al. reported that nelfinavir treatment increased ER stress markers in breast and ovarian cancer cells [21,39]. More recently, Mahammeed et al. reported that nelfinavir was highly effective to inhibit the growth of HCC cells in vitro and in vivo when combined with the PERK inhibitor ISRIB, which is an experimental drug that inhibits the integrated stress response (ISR); the ISR is a term that encompasses the phosphorylation of eIF2α not only by PERK, but also by other kinases including PKR, GCN2 and HRI [59]. Combined treatment of ISRIB and ER stressor nelfinavir cooperatively inhibited maturation and phosphorylation of specific receptor protein tyrosine kinases such as c-MET and EGFR, likely via selective sequestration of these receptors in the ER.

At the downstream level of nelfinavir-mediated ER stress, ATF4 inhibited the activity of mammalian target of rapamycin (mTOR) by activating sestrin-2 (SESN2) protein, which contributed to the inhibition of protein translation [43]. In TNBC cells, dual treatment by ER stress-aggravating compounds, nelfinavir and celecoxib, resulted in increased levels of GRP78, ATF3, and CHOP, which were further enhanced when the autophagy inhibitor chloroquine was added to the combination [41]. Chakravarty et al. suggested that nelfinavir sensitizes doxorubicin-resistant breast cancer cells back to doxorubicin via upregulation of ER stress proteins ATF4 and CHOP. ATF4 and CHOP further upregulated a death sensor, tribbles homolog-3 (TRIB-3), which inhibited Akt phosphorylation and activated the apoptotic pathway facilitating chemosensitization [60]. Mathur et al. further demonstrated ER stress and TRIB-3-mediated chemosensitization of castration-resistant prostate cancer cells to docetaxel during combination of nelfinavir and curcumin [46]. In liposarcoma and prostate cancer cells, nelfinavir led to the accumulation of sterol regulatory binding protein-1 (SREBP-1) and ER stress protein ATF6 [61,62]. SREBP1 is a key regulator of adipocyte differentiation and lipid synthesis in cells [63]. Both SREBP-1 and ATF6 are ER-resident transcription factors which are translocated and cleaved in the Golgi apparatus by site-1 protease (S1P) and site-2 protease (S2P) in a process named regulated intramembrane proteolysis (RIP) to release the active transcription factors [64]. Guan et al. mechanistically demonstrated that the nelfinavir-mediated accumulation of SREBP-1 and ATF6 in prostate cancer cells were the outcome of inhibition of the enzyme S2P by the drug [65]. Additionally, nelfinavir and nelfinavir analogues increased the level of GRP78 in prostate cancer cells and decreased the level of the SREBP-1 target enzyme fatty acid synthase (FAS). At the mRNA level, a time-dependent increase in ER stress-related genes ATF6, GRP78, and XBP-1 was observed during treatment by nelfinavir in castration-resistant prostate cancer cells [65].

Protein translation machineries are exploited by tumor cells to generate oncogenic signals; as such, targeting the components of protein translation can be beneficial to halt tumor growth [66]. De Gassart et al. mechanistically demonstrated that nelfinavir could inhibit protein translation in two possible ways—by inhibiting translation initiation and elongation [66]. Nelfinavir was shown to activate the eukaryotic elongation factor 2 kinase (eEF2K) and interfere with protein translation by phosphorylating and inhibiting the eukaryotic elongation factor 2 (eEF2) [67]. The authors further observed that nelfinavir promoted phosphorylation of eIF2α and activated downstream ATF4, CHOP, and growth arrest and DNA damage inducible protein 34 (GADD34) in cervical cancer cells and mouse embryonic fibroblasts (MEFs) [68]. Initial phosphorylation of eIF2α inhibits global synthesis of proteins, however, in case of prolonged and irreversible proteotoxic damage, activated GADD34 recruits protein phosphatase 1 and dephosphorylates eIF2α to restart protein translation and facilitate the synthesis of proteins necessary for cell death.

Hyperactivated mTOR complex has been implicated in many cancers, mostly as a consequence of the inhibition of the upstream regulator protein tuberous sclerosis complex (TSC). Loss-of-function mutations in Tsc1 or Tsc2 withdraw the inhibitory action over mTOR and lead to excessive and aberrant protein synthesis [69]. Johnson et al. demonstrated that basal ER stress was elevated in Tsc2^−/−^ MEFs, which was further aggravated by nelfinavir, evidenced from increased mRNA level of CHOP and spliced XBP-1 and increased protein levels of GRP78 and IRE1α [69]. The authors also observed that nelfinavir-mediated increase in mRNA and protein levels of ER stress markers were further increased by the addition of bortezomib in Tsc2^−/−^ mTOR-hyperactive cells. Dual therapy by nelfinavir and bortezomib resulted in increased expression of CHOP and ATF4 in the tumors derived from xenograft models of mTOR hyperactive cells [70]. Moreover, combined therapy of nelfinavir and mefloquine (an analogue of chloroquine) or salinomycin (an anti-cancer antibiotic) resulted in activation of the ATF4-CHOP-GADD34 arm of the ER stress pathway in Tsc2^−/−^ mTOR-hyperactive cells [71,72]. Tian et al. also reported phosphorylation of eIF2α and increased protein levels of ATF4 and CHOP in response to nelfinavir in glioblastoma cells [52]. Phosphorylation of eIF2α was also reported in nelfinavir-treated pediatric refractory leukemia cells [44]. In renal cancer cells, ER stress was shown to be induced by nelfinavir, as evidenced by the increase in GRP78, endoplasmic reticulum resident protein 44 (ERp44), and endoplasmic oxidoreductin-1-like protein α (ERO1-Lα) [28]. Dual treatment by nelfinavir and HDAC inhibitor panobinostat also activated ER stress in renal cancer cells [29]. Sato et al. demonstrated aggravation of ER stress during combined treatment of nelfinavir and ritonavir in bladder cancer cells evidenced from increased GRP78, ERp44, and ERO-1Lα [27].

Kawabata et al. reported that dual therapy of nelfinavir and bortezomib involves ER stress induction and aggravation of proteotoxic stress in NSCLC and leukemia cells. The authors observed that a dose of 10 μM nelfinavir was not sufficient to activate caspases; however, combined treatment of nelfinavir and bortezomib induced strong cleavage of caspases-8, 9, 3, and 7, with subsequent cleavage of the downstream effector PARP. Inhibition of protein translation by cycloheximide reduced the percentage of dead cells during combination therapy of nelfinavir and bortezomib, suggesting the necessity of proteotoxic pressure for apoptosis [56]. This concept was further demonstrated in malignant glioma cells during nelfinavir monotherapy and in renal cancer cells during combination therapy of nelfinavir and panobinostat. In both instances, inhibition of protein synthesis by cycloheximide rescued the cells from nelfinavir-induced cell death [29,58]. Pyrko et al. also observed ER stress-related death in nelfinavir-treated malignant glioblastoma cells, which was associated with activation of ER stress-related caspase-4 [58]. Kraus et al. observed ER stress-related death and caspase-4 activation in MM cells during the combination of nelfinavir and bortezomib. Furthermore, nelfinavir showed higher synergistic lethal potency with bortezomib and carfilzomib than other HIV-PIs in MM cells and facilitated overcoming bortezomib and carfilzomib resistance [73]. Likewise, in leukemia cells, nelfinavir reduced viability alone and in a synergistic manner when combined with bortezomib [74]. Bruning et al. observed a similar synergistic cell death by dual treatment of nelfinavir and bortezomib in cervical cancer cells with activation of ER stress proteins GRP78 and ATF3 [23].

Activation of ER stress following chemotherapy with nelfinavir as a single agent or in combination with other chemotherapy, such as bortezomib, has been reported in patient samples. Blumenthal et al. reported phosphorylation of eIF2α at serine 51 and enhanced levels of CHOP and ATF3 in the peripheral blood mononuclear cells (PBMCs) of patients receiving nelfinavir to treat solid cancer at the maximum tolerated dose of 3125 mg twice daily [57]. Driessen et al. reported increased GRP78, CHOP, and ER stress-related protein disulfide isomerase (PDI) in PBMCs of MM patients receiving both nelfinavir and bortezomib [75]. Hitz and colleagues observed enhanced levels of CHOP and IRE1α in PBMCs of lenalidomide-refractory MM patients receiving combination therapy of nelfinavir, lenalidomide, and dexamethasone [76].

Morphologically, vacuolization and expansion of the ER have been demonstrated in ER stress-activated cells treated with nelfinavir. Bruning et al. reported increased vacuolization of the cytoplasm in ovarian cancer cells following treatment with nelfinavir, which colocalized with ER-resident proteins and GRP78 observed through immunofluorescence microscopy [21]. Gills et al. also observed nelfinavir-mediated vacuolization in lung cancer cells, which colocalized with immunofluorescent aggregates containing ER-targeted sequence of calreticulin [19]. Mahoney and colleagues reported ER swelling following the treatment of nelfinavir in chronic lymphocytic leukemia cells with increased accumulation of fluorescent calnexin protein, suggesting accumulation of misfolded ER proteins [77]. In glioblastoma cells, Pyrko et al. observed, through transmission electron microscopy, swelled ER cisternae during nelfinavir treatment [58]. Kawabata et al. observed that nelfinavir-mediated vacuolization was reduced during treatment with protein synthesis inhibitor cycloheximide [56]. Notably, cycloheximide treatment inhibited the cytotoxicity towards renal cancer cells receiving a combination of panobinostat and nelfinavir, underscoring the role of protein overload in nelfinavir-associated toxicity [29].

### 2.4. Autophagy

Autophagy is an evolutionarily conserved catabolic process involved in digesting misfolded proteins or cellular organelles, and recycling cellular compounds or macromolecules to overcome energy and nutrient deprivation [78]. Different assays are utilized to assess the autophagic status of cells, among which tracking the expression of the microtubule-associated ubiquitin-like light-chain protein 3 (LC3) is performed frequently [79]. In immunoblots, endogenous LC3 is visualized as two protein bands: a cytosolic component LC3I and a membrane-bound phosphatidylethanolamine (PE) conjugated component LC3II. The membrane-bound LC3II is a component of autophagosomes, and its enhanced expression indicates an increase in their numbers. Increased autophagosomes, however, may be the outcome of either acceleration of the autophagic process or impairment of lysosomal activity. Thus, enhanced LC3II at a given time point may not readily indicate an increased rate of autophagy. Instead, increased rate of autophagy is assessed by measuring autophagic flux. To determine the autophagic flux, the expression level of LC3II is studied in the presence of a lysosome inhibitor; an additive increase in LC3II expression in the presence of a lysosome inhibitor such as bafilomycin A1, will indicate increased flux in contrast to a lack of change in LC3II expression—which will indicate lysosome impairment. Alternatively, time-sensitive tracking of degradation of p62—a ubiquitinated substrate of autophagy—can confirm autophagic flux [79]. Visualization of autophagosomes under the electron microscope, measurement of LC3 labelled puncta through immunofluorescence, and measurement degradation of green fluorescent protein (GFP) labelled LC3 by FACS are also ways to determine the autophagic status of cells [78].

Gills et al. described the autophagy inducing properties of nelfinavir in NSCLC cells. The authors observed an increase in the membranous form of LC3, known as LC3II, suggesting increased synthesis of autophagosomes [19]. Nelfinavir also increased the GFP labelled fluorescent LC3 aggregates, which was abrogated by the addition of autophagy inhibitor 3-MA. Transmission electron microscopy of nelfinavir treated human oral squamous cell carcinoma H157 cells revealed evidence of organelle containing degradative autophagosomes. The authors linked ER stress as an upstream activating factor of autophagy and concluded that the induced autophagy could be a compensatory survival mechanism, as the inhibition of autophagy by 3-MA resulted in enhanced cytotoxicity [19]. Enhanced apoptosis due to combined treatment of nelfinavir and 3-MA was also observed in refractory pediatric leukemia cells [44]. Gill et al. later highlighted four possible mechanisms by which nelfinavir could exert its autophagy-inducing properties. Firstly, nelfinavir-mediated mTOR inhibition could be linked with autophagy as a consequence of transient Akt inhibition. Secondly, ER stress induced by nelfinavir likely induces pro-survival autophagy through phosphorylation of eIF2α and increased expression of ATF4. Thirdly, enhanced eukaryotic elongation factor 2 kinase (eEF2K)-mediated phosphorylation of elongation factor 2 (EF2) by nelfinavir possibly activates autophagy. Finally, nutrient starvation resulted from the blockade of growth factor receptor signaling by nelfinavir can promote autophagy [80]. Bruning et al. reported that nelfinavir increased the expression of the autophagosome marker LC3II in estrogen receptor-negative breast cancer cells [39]. They also demonstrated that nelfinavir promoted ATF4 driven SESN2 expression in different cells. SESN2 inhibits the mTOR complex—a master downregulator of cellular autophagy. Thus, by upregulating SESN2, nelfinavir enhanced the formation of autophagosomes, which were visualized by fluorescent microscopy using an autophagic vesicle detection marker [43]. Guan et al. suggested enhanced autophagy by quantifying the turnover of GFP labelled LC3, utilizing FACS in nelfinavir treated androgen-dependent and castration-resistant prostate cancer cells [62]. Escalante et al. reported reduced co-localization of LC3II and LAMP2 (a lysosomal marker) during nelfinavir monotherapy and in combination with bortezomib in MM cells, suggesting impaired autophagy—likely due to impaired fusion of autophagosomes and lysosomes. The authors further observed a decrease in the level of calpain activity following nelfinavir treatment in MM cells [81]. Calpains are Ca^2+^-dependent cysteine proteases involved in the cleavage of cytoskeletal proteins, signal transducers, and membrane receptors [82]. Calpain deficiency has been shown to be involved in impaired autophagy and activation of the apoptotic switch [82], which was suggested to be a reason for the synergistic lethal interaction between bortezomib and nelfinavir in MM cells [81]. Kushchayeva et al. observed enhanced expression of LC3II in nelfinavir treated medullary thyroid cancer cells with a concomitant degradation of lysosomal substrate p62, indicating an increase in the autophagic process [38]. A similar outcome of enhanced LC3II and decreased p62 was observed in mTOR hyperactive tumorigenic mouse embryonic fibroblast cells—lacking tuberous sclerosis gene (Tsc2^−/−^)—during nelfinavir monotherapy [69]. In a multidrug-resistant (MDR) breast cancer model (MCF-7/Dox), it was observed an increase in LC3II during combined treatment with nelfinavir and doxorubicin [60]. In nelfinavir-treated cisplatin-sensitive ME-180 and cisplatin-resistant (CPR) ME-180 cervical cancer cells, LC3II was also increased [42]. Increased LC3II expression was also seen in PBMCs of nelfinavir/lenalidomide/dexamethasone-treated lenalidomide-refractory MM patients [76].

Beclin-1 is a critical regulator of autophagy at the early stage, and changes in beclin-1 expression is monitored to assess the autophagic status in cells [80]. However, change in beclin-1 was not observed during nelfinavir monotherapy [38,44] or combined therapy with other autophagy inhibitors [41]. Gills et al. opined that nelfinavir-mediated autophagy may be beclin-1 independent [80].

Autophagy is generally known as a pro-survival mechanism; thus, it has been hypothesized that inhibiting the pathway may provide benefits by aggravating cytotoxicity during cancer therapy. To explore this hypothesis, nelfinavir has been tested in combination with autophagy inhibiting drugs to induce heightened cytotoxicity in the cancer cells. Enhanced cytotoxicity due to the combination of nelfinavir and a class III PI3K and autophagy inhibitor 3-MA in NSCLC and pediatric leukemia cells has been described before, where 3-MA resulted in reduced LC3II [19,44]. A widely used anti-malarial drug chloroquine is an inhibitor of late-stage autophagy and has been used in combination with nelfinavir to demonstrate enhanced cytotoxicity in chronic lymphocytic leukemic cells [77], tuberous sclerosis negative (Tsc2^−/−^) MEF cells, and human lung cancer cells (NCI-H460) [69]. Thomas et al. reported that chloroquine further increased the cytotoxicity of dual treatment of nelfinavir and DMC selectively in TNBC cells [41].

As stated earlier, bafilomycin A1 is an inhibitor of autophagy; it works via inhibition of v-ATPase transporter—preventing entry of protons in lysosomes; thereby, it decreases acidification and functionality of lysosomes. According to Johnson et al. the combination of nelfinavir and bafilomycin-A1 did not induce cytotoxicity to the same extent as the combination of nelfinavir and chloroquine derivatives in Tsc2^−/−^ MEFs. Furthermore, autophagy was not suppressed during the combination of nelfinavir and chloroquine-derivative mefloquine in Tsc2^−/−^ cells, which implies that mechanisms other than autophagy may be involved while inducing cytotoxicity by combining putative autophagy-inhibitors of different chemical natures with nelfinavir [69]. Autophagy-inhibitor mefloquine was shown to enhance nelfinavir-mediated cytotoxicity in breast cancer (MCF7), colon cancer (HCT116), lung cancer (NCI-H460), and Tsc2^−/−^ cells. The cytotoxicity induced by the combined treatment of mefloquine and nelfinavir was rescued by the addition of methyl pyruvate, indicating energy deprivation as a possible mechanism of the heightened cytotoxicity [70]. Collectively, the reports suggest that nelfinavir can modulate the autophagic process in cancer cells in a cell type-specific manner.

### 2.5. Inhibition of the Proteasome

The proteasome is a cytoplasmic and nucleoplasmic high-molecular-weight structure geared towards degrading proteins—tagged with ubiquitin or other ubiquitin-like molecules—to maintain cellular proteostasis [83]. The 26S proteasome contains a cylindrical catalytic 20S core, which is capped on each end by 19S regulatory components. The 20S catalytic core is comprised of α and β subunits, among which β subunits are responsible for specific proteolytic activities: β1/β1i for caspase-like, β2/β2i for trypsin-like, and β5/β5i for chymotrypsin-like activities. The first-generation proteasome inhibitor bortezomib constitutes the mainstay treatment for MM. Second-generation proteasome inhibitors, such as carfilzomib, are also available with demonstrated lesser neurotoxicity [84].

Nelfinavir was shown to affect the proteasome in selective cancer cell lines. Bono et al. reported that nelfinavir inhibited the chymotrypsin-like activity of the proteasome of MM cells (U266) and showed enhanced ubiquitination in immunoblots—a surrogate marker of proteasome inhibition—of U266 cells treated for 24 h with 5 μM nelfinavir [37]. Driessen et al. demonstrated a moderate decrease in the β2 and β1/β5 activities of the proteasome after nelfinavir treatment in PBMCs of patients having refractory-MM and other hematologic cancers [75]. Bortezomib targets the β5 subunit of the proteasome and inhibits protein degradation [85]. In contrast, enhanced β2 activity of the proteasome is associated with bortezomib resistance in MM patients, whereas a concomitant decrease in β2 activity during bortezomib treatment can confer re-sensitization to bortezomib [75,86]. Indeed, a combination of nelfinavir and bortezomib showed a positive response in bortezomib refractory cancer [20,75]. Kraus et al. also demonstrated proteasome inhibitory effects of nelfinavir on bortezomib-resistant MM cell lines in vitro, where nelfinavir showed expected bortezomib sensitizing effects [73]. The same group also demonstrated that the proteasome inhibitory properties of nelfinavir on acute myeloid leukemia (AML) cells specially contributed to the cytotoxic effects of the drug [74]. Kawabata et al. observed proteasome inhibitory effects of nelfinavir on MM (RPMI8226) and NSCLC (H157) cells indicated by enhanced ubiquitination via immunoblot. Although the ubiquitination was moderate during nelfinavir monotherapy, it was considerably enhanced when combined with bortezomib, suggesting a synergistic interaction [56].

Combined treatment of nelfinavir and second-generation proteasome inhibitor carfilzomib was shown to re-sensitize carfilzomib-resistant MM cells to carfilzomib-mediated cytotoxicity and facilitated re-inhibition of proteasome subunits. The reason for nelfinavir-mediated carfilzomib re-sensitization was attributed to the ability of nelfinavir to inhibit the expression of ABCB1—a multidrug-resistant efflux pump—resulting in the reduced efflux of intracellular carfilzomib [87]. Pyrko et al. showed nelfinavir-driven enhanced ubiquitination in glioblastoma cells (U251) indicating proteasome inhibition, which was reversed by the use of protein synthesis inhibitor cycloheximide [58]. Similarly, proteasome inhibition through the dual treatment of nelfinavir and bortezomib was decreased by the addition of cycloheximide [56].

Conversely, some studies reported a non-inhibitory effect of nelfinavir on the proteasome. Escalante et al. did not observe a decrease in the chymotrypsin-like activity of the proteasome in response to a pharmacologically relevant dosage of nelfinavir (10 μM) in MM cells; however, it did not hinder the synergistic cytotoxic effect of bortezomib and nelfinavir [81]. Bruning et al. did not observe, in response to nelfinavir, any change in the chymotrypsin, trypsin, or caspase-like activities of the proteasome in cervical cancer cells and human B-lymphoblastoid cells [23], or any decrease in the chymotrypsin-like activity of the proteasome in breast cancer cells [39]. Moreover, Sato et al. observed an unexpected reduction in the accumulation of ubiquitinated proteins during dual therapy of nelfinavir and ritonavir in bladder cancer cells [27]. Jiang et al. reported that nelfinavir promoted the degradation of CdC25A phosphatase—a substrate of the proteasome—in melanoma cells and addition of the proteasome inhibitor MG-132 halted the degradation. This phenomenon, in fact, suggests an enhancement of proteasome activity in response to nelfinavir [25].

One possible explanation of the discrepancy in study findings reporting the proteasome inhibitory function of nelfinavir is that the mechanism of action could be diverse and cell type specific. It is important to address whether nelfinavir targets the mature 26S proteasome or the free 20S subunit, which may not be fully efficient to impair overall proteasome activity. According to Bono et al., nelfinavir decreased the 26S proteasome activity in MM cells [37], while other studies reported that nelfinavir targeted the 20S proteasome in breast cancer [88], and head and neck cancer cells [89].

Importantly, it has been reported that the mammalian 20S proteasome can cleave the same site targeted by the HIV proteases in HIV [1]. Pajonk et al. reported inhibition of the 20S proteasome by the HIV-PI saquinavir in non-HIV associated cancer cells, which was associated with apoptosis and radio-sensitization [90]. Piccinini reported that nelfinavir and saquinavir decreased both the 26S and 20S proteasome activity in human red blood cells [91]. Recently, Fassmannová et al. proposed that nelfinavir can inhibit proteasome synthesis by inhibiting the transcription factor TCF/Nrf1. Reactivation of TCF/Nrf1 during treatment with proteasome inhibitors results in increased proteasome synthesis—known as the bounce-back response—eliciting resistance to proteasome inhibitors in MM [92]. Nelfinavir possibly inhibits the translation and maturation of TCF/Nrf1, leading to the repression of re-synthesis of the proteasome, which can explain the better outcome in clinical trials administering nelfinavir in bortezomib refractory MM [20,76]. The study by Fassmannová et al. [92] further elicits the possibility that the proteasome inhibitory property of nelfinavir may not be due to the direct repression of the proteasome subunits, but rather via an indirect phenomenon.

### 2.6. Signal Transduction Pathways

Aberrant signaling pathways are common in cancers and dampening atypical signaling feedback—developed through mutations in the components of the signaling cascades—is a well-established pharmacological strategy against cancer. Multiple studies have demonstrated that nelfinavir can target different cellular signaling pathways. The primary intracellular target of nelfinavir—responsible for its anti-cancer properties—has not yet been identified definitively; however, some groups suggested heat shock protein 90 (HSP90) as a putative primary target through in silico and in vitro methods. Arodola et al. suggested nelfinavir as a more potent binding molecule for HSP90 than other HIV-PIs, through homology modeling, molecular docking simulation, and analysis of binding affinity [18]. Shim et al. demonstrated selective anti-tumor activity of nelfinavir in human epidermal growth factor receptor 2 (HER2)-positive breast cancer cells [88]. To identify the molecular target, the authors conducted a genome-wide screening of nelfinavir using haploinsufficiency yeast strains, which revealed HSP82—the yeast orthologue of mammalian HSP90—to be a possible binding partner. Co-immunoprecipitation and trypsin digestion profiling in mammalian cells indicated that nelfinavir might affect HSP90 in a different manner than known HSP90 inhibitors, e.g., geldanamycin and novobiocin. Nelfinavir also decreased the protein level of HSP70 and HSP90 in HER2-positive breast cancer cells, which may have contributed to interrupted protein folding leading to ER stress—suggested from the enhanced phosphorylation of eIF2α [88]. Kuschayeva et al. reported an increase in the level of HSP90 protein in patient samples of hereditary thyroid medullary carcinoma, which was associated with significant metastasis and *RET* mutation [38]. Although the authors did not observe a change in the protein level of HSP90 in response to nelfinavir in *RET*-mutated thyroid cancer cells in vitro, the signaling of HSP90 client proteins—E-cadherin, tyrosine kinase *Src* (SRC) and connexin-34—was downregulated, suggesting nelfinavir-mediated post-translational modification of HSP90 [38]. Mutation of the proto-oncogene *RET* is common in medullary thyroid cancer, and RET protein is a substrate to HSP90-mediated protein folding and processing. Mutant RET can exploit HSP90 for stability, and inhibition of HSP90 can be used as a potential strategy to induce 26S proteasome-mediated degradation of wild type and mutant RET [93,94]. In medullary thyroid cancer cells, nelfinavir decreased the expression of RET and its downstream signaling effectors Akt, ERK1/2 and p70S6K [38].

Akt is an important client protein of HSP90, and Soprano et al. demonstrated that nelfinavir promotes dissociation of HSP90-Akt complex without affecting the total Akt at the mRNA and protein level in breast cancer cells [30]. Nelfinavir was shown to decrease both phosphorylated and total levels of Akt in breast cancer cells, along with decreased expressions of downstream proteins of the Akt signaling cascade [30]. Decreased phosphorylation of Akt client proteins PRAS40, FOXO3a and Bad was also seen in mTOR hyperactivated (*Tsc2*^−/−^) cells in response to nelfinavir monotherapy and in combination with salinomycin [71]. Shim et al. reported decreased phosphorylation of Akt and ERK1/2 in response to nelfinavir in HER2-positive and -negative breast cancer cells. In HER2-positive breast cancer cells, nelfinavir dissociated the interaction between HSP90 and HER2 and downregulated total protein levels of Akt and HER2 [88]. Decreased Akt phosphorylation in response to nelfinavir was also evident in MM [37,73], AML [74], pediatric refractory leukemia [44], diffuse B-cell lymphoma [95], doxorubicin-resistant breast cancer [60], prostate cancer [46,48], and NSCLC [19,47].

Downregulation of Akt signaling has been a widely mentioned effect of nelfinavir in cancer cells and has been proposed as a radiosensitizing strategy [96,97,98]. Chronic usage of nelfinavir in HIV infected patients, results in impaired glucose metabolism, insulin resistance, and lipodystrophy, which suggests a probable role of nelfinavir via inhibition of the PI3K–Akt–mTOR pathway. Gupta et al. demonstrated Akt dephosphorylation and radiosensitization by nelfinavir in bladder cancer and head and neck carcinoma cells and animal models [96]. The authors further suggested that nelfinavir works mechanistically via proteasome inhibition leading to the activation of the UPR, which forms and activates the phosphatase complex PP1/GADD34 responsible for dephosphorylating eIF2α and Akt [89]. Infection by human papillomavirus (HPV) has been associated with better response to radiation in head and neck cancer; Gupta and colleagues showed that nelfinavir sensitized both HPV infected and non-infected head and neck carcinoma cells to radiation with a concomitant decrease in phosphorylated Akt [99]. Jiang et al. demonstrated that glioblastoma cells lacking wild type phosphatase and tensin homologue (PTEN) are resistant to radiation and temozolomide, which can be overcome by nelfinavir. Nelfinavir-mediated radiosensitization in PTEN-deficient glioblastoma cells was associated with decreased phosphorylation of Akt [100]. Kimple and colleagues showed that *KRAS* mutation confers resistance to radiation in pancreatic cancer cells likely due to failure to downregulate Akt phosphorylation. Both nelfinavir and the PI3K inhibitor LY294002, decreased phosphorylation of Akt in pancreatic cancer cells expressing either wild type or mutant *KRAS*, and sensitized them to radiation [101]. Cuneo and colleagues observed decreased angiogenesis in response to nelfinavir, which was associated with decreased Akt phosphorylation in endothelial cells. Additionally, the combination of nelfinavir and radiation showed an additive effect in decreasing angiogenesis in a mouse xenograft tumor model of Lewis lung carcinoma [102]. Nelfinavir-mediated reduction in vascular endothelial growth factor (VEGF) and hypoxia-inducible factor 1α (HIF1α) through inhibition of the PI3K–Akt pathway has been observed by Pore et al. in head and neck carcinoma, lung cancer, and glioblastoma cells which contributed in reduced angiogenesis and potentiation of radiotherapy [103,104]. Potentiation of radiotherapy via nelfinavir was also demonstrated in pituitary adenoma cells, which was associated with reduced phosphorylation of ribosomal S6 protein—a downstream effector of PI3K–Akt–mTOR cascade [105].

Plastaras et al. observed a decreased level of Akt phosphorylation in PBMCs of HIV-infected patients treated with nelfinavir and saquinavir. The authors suggested that the level of phospho-Akt in PBMCs could be used as a surrogate biomarker to assess pharmacological efficacy in targeting Akt signaling by HIV-PIs [106]. Blumenthal and colleagues reported anti-tumor activity of nelfinavir in patients with solid tumors, which was associated with decreased phospho-Akt in PBMCs [57]. Similarly, Brunner et al. reported the radiosensitizing effects of nelfinavir in patients having locally advanced pancreatic cancer, with associated decreased level of phosphorylated Akt in PBMCs of the treated patients [107].

Nelfinavirmediated a decrease in Akt phosphorylation with concomitant anti-tumor activities—observed through cell culture experiments—that has not always been translated in vivo and in clinical trials. In one study, nelfinavir was effective in eliciting anti-cancer effects in the adenoid cystic carcinoma cells, which was associated with Akt dephosphorylation, justifying usage of nelfinavir in clinical trials [108]. However, Hoover et al. did not observe a meaningful positive outcome in a phase II clinical trial testing the beneficial effects of nelfinavir in patients having adenoid cystic carcinoma [109]. Moreover, Leibscher et al. reported nelfinavir-mediated downregulation of phosphorylation of Akt at the Ser473 position in PC-3 prostate cancer cells; however, nelfinavir failed to improve the efficacy of radiation therapy in prostate cancer in vivo [110]. Gills and colleagues reported decreased phosphorylation of basal and growth factor activated Akt in response to nelfinavir in lung cancer cells; however, nelfinavir-mediated reduction in Akt phosphorylation was not evident in tumor samples from xenograft models of lung cancer cells. Of notice, despite the discrepancy in Akt phosphorylation status, the anti-tumor efficacy of nelfinavir against lung cancer cells was similar both in vitro and in vivo [19]. Tumor growth impairment by nelfinavir in xenograft models of HER2-positive breast cancer cells was not associated with reduced phosphorylation of Akt, although decreased Akt phosphorylation by nelfinavir in HER2-positive breast cancer cells was evident in vitro [88]. In contrast, activation of Akt has been reported in estrogen receptor-negative breast cancer cells and melanoma cells during short term treatment of nelfinavir, which did not hamper the antiproliferative effects of the drug [25,39].

To date, no evidence pointed at a direct interaction of nelfinavir with Akt; however, modulation of Akt in response to nelfinavir indicates upstream signaling activity. Xie et al., based on computational prediction and kinase assays, proposed binding of nelfinavir to 51 off target protein kinases, the majority of which belong to the tyrosine kinase, cAMP-dependent, cGMP-dependent, and protein kinase C families—suggesting broad spectrum poly-pharmacological role of nelfinavir, i.e., the possibility of binding of nelfinavir with multiple targets with varying affinity [17,111]. Gills and colleagues demonstrated reduced activation of epidermal growth factor receptor (EGFR) and insulin-like growth factor receptor (IGFR) in response to nelfinavir, leading to downstream inactivation of Akt in NSCLC cells [19].

Nelfinavir has also been demonstrated to target other proliferative signaling cascades. Downregulation of the mitogen-activated protein kinase pathway (MAPK)—by decreased phosphorylation of ERK—in response to nelfinavir has been reported in medullary thyroid cancer [38], adenoid cystic carcinoma [108], MM [37,112], and breast cancer cells [88]. However, decreased phosphorylation of ERK in cancer cells is not a universal response to nelfinavir treatment, as nelfinavir did not downregulate ERK phosphorylation in NSCLC [47], pancreatic cancer [101], and pituitary adenoma [105]. Downregulation of phospho-ERK in response to nelfinavir was further observed during combination with doxorubicin in doxorubicin-resistant chronic myeloid leukemia cells [45], and with bortezomib against MM cells [73]. Nelfinavir sensitized BRAF-mutated melanoma cells to MEK inhibitors and BRAF inhibitors via SMAD-mediated downregulation of PAX and MITF, and decreased phosphorylation of ERK during combination with inhibitors of MEK or BRAF [113]. Conversely, Bruning et al. reported enhanced phosphorylation of ERK in ovarian cancer and cervical cancer cells carrying wild type p53, which possibly led to the activation of anti-apoptotic Mcl-1 protein [23,35]. Enhanced ERK phosphorylation was also reported during the combination of nelfinavir and tamoxifen in estrogen receptor-negative breast cancer cells [39].

Decreased phosphorylation of signal transducer and activator of transcription 3 (STAT3) in response to nelfinavir was observed in MM [37,112] and prostate cancer [48]. Nelfinavir has also shown to inhibit HDAC [27] and has been shown to synergize with the HDAC inhibitors panobinostat [29] and valproic acid [37]. Of note, inhibition of HDAC6 by nelfinavir leads to enhanced ER stress following inhibition of HSP90 through acetylation leading to protein misfolding, which suggests HDAC inhibitors as potential ER stress aggravating chemotherapeutic agents [114].

Nelfinavir has also been suggested to be involved in altering metabolic signaling. Depletion of ATP has been reported in nelfinavir-treated doxorubicin-resistant chronic myeloid leukemia cells, which can be restored by the addition of exogenous glucose, resulting in the withdrawal of nelfinavir-mediated sensitization to doxorubicin. Metabolic stress incurred by nelfinavir results in the activation of 5′-AMP-activated protein kinase (AMPK) [27,38,69]. AMPK leads to inhibition of mTOR and activates autophagy at the downstream level, facilitating synergism between nelfinavir and autophagy inhibitors, such as chloroquine [69]. Activation of AMPK and downregulation of mTOR also occurred following dual treatment of nelfinavir and salinomycin or mefloquine [71,72]. The addition of energy substrate methyl pyruvate inhibited nelfinavir- and mefloquine-mediated AMPK activation and rescued from cell death [72]. Nelfinavir promotes inhibitory phosphorylation of eEF2 through eEF2K, which leads to the arrest of protein synthesis [66,67]. Nelfinavir-driven activation of eEF2K may or may not be dependent on AMPK [67,80]. Nelfinavir can also inhibit mTOR via activation of ATF4-mediated SESN2, which can also lead to metabolic stress and autophagy [43].

### 2.7. Oxidative Stress and Mitochondria

Regulated production of ROS is crucial for critical cellular functions such as cell growth, differentiation and apoptosis, by promoting oxidative modification of proteins involved in these pathways. However, high production of ROS is detrimental to the cells as it induces damage to the DNA, proteins, and lipids. It has been demonstrated that cancer cells tend to produce excess ROS and have a higher level of basal oxidative stress than normal cells, which suggests a therapeutic benefit of aggravation of oxidative stress through pharmacological intervention, leading to selective cytotoxicity in cancer cells [115]. Anti-cancer properties exerted by nelfinavir have been linked to enhanced oxidative stress. Bruning et al. demonstrated that nelfinavir reduced the level of the intracellular antioxidant glutathione (GSH) in TNBC cells in a dose-dependent manner. Nelfinavir-mediated enhanced oxidative stress contributed to reduced cell viability, which was rescued by the addition of exogenous antioxidants—GSH or NAC [39]. Kushchayeva et al. showed that nelfinavir reduced the mitochondrial membrane potential in medullary thyroid cancer cells in a dose-dependent manner, with a concomitant increase in γH2AX—a marker of DNA damage. Enhancement of γH2AX was mitigated by the addition of exogenous antioxidant NAC, indicating a direct cytotoxic role of nelfinavir-induced oxidative stress in these cells. In a comparative gene expression study, both nelfinavir and hydrogen peroxide (H_2_O_2_) induced the expression of genes regulating the production of superoxide [116]. Liu and colleagues corroborated nelfinavir-mediated depolarization of the mitochondrial membrane in doxorubicin-resistant CML cells, which resulted in a loss of adenosine triphosphate (ATP)—suggesting induction of metabolic stress. The authors further observed an increase in ROS level during the combination of suboptimal doses of doxorubicin and nelfinavir in doxorubicin-resistant CML cells [45]. Xiang et al. showed enhanced intracellular and mitochondrial ROS production in cervical cancer cells; nelfinavir-mediated cellular apoptosis was rescued by the addition of antioxidant NAC and a mitochondria-targeting superoxide and alkyl-radical scavenger Mito-TEMPO, which indicates the role of oxidative stress in nelfinavir-induced cytotoxicity [33]. Xia et al. demonstrated that nelfinavir, combined with metformin, induced ROS production in cervical cancer cells, with a concomitant increase in NAD-dependent deacetylase sirtuin-3 (SIRT3), which is a primary mitochondrial acetyl-lysine deacetylase required to maintain energy homeostasis in the electron transport chain [34,117,118]. Besse et al. showed nelfinavir and lopinavir-mediated ROS production in carfilzomib-resistant MM cells, which was rescued by the addition of mitochondrial permeability transition pore antagonist decylubiqinone [87].

### 2.8. Tumor Microenvironment

Nelfinavir plays a role in modulating the tumor microenvironment by inhibiting abnormal angiogenesis, improving oxygenation of the tumor tissue, inhibiting the growth of tumor stem cells, reducing the release of matrix metalloproteinases, and inhibiting invasion. Pore et al. demonstrated decreased expression of VEGF in head and neck squamous cell carcinoma, lung cancer and glioblastoma cells in response to nelfinavir, which was associated with reduced angiogenesis in vivo [103,104]. Nelfinavir-mediated reduction in VEGF was attributed to decreased phosphorylation of Akt and the transcription factor SP1, and further reduction in HIF1α. Of note, both SP1 and HIF1α can bind to the promoter region of VEGF and transactivate the gene [103]. Functionally, decreased VEGF and HIF1α was associated with increased radiosensitivity during treatment with nelfinavir in vivo. The authors further observed a decrease in the hypoxia marker EF5 in nelfinavir treated tumors, which suggested increased tissue oxygenation despite reduced angiogenesis. As previous studies associated improved tissue oxygenation with radiosensitization [119], Pore et al. speculated that the reduction in VEGF might have led to the normalization of the vascular bed and a reduction in abnormal vessels formation, which promoted better tissue oxygenation and enhanced radiosensitivity [103]. Cuneo et al. demonstrated in vitro that nelfinavir—alone or in combination with radiotherapy—inhibited the growth of human umbilical vein endothelial cells (HUVECs), and reduced cell migration and invasion. Potent reduction in angiogenesis was also evident in a xenograft model of lung carcinoma in response to the combination of nelfinavir and radiotherapy [102]. Qayum et al. demonstrated that nelfinavir altered the abnormal phenotype of the tumor vasculature by decreasing vessel tortuosity and showed physical similarity with the normal vascular system in xenografts of EGFR-mutated cells having constitutively active PI3K–Akt signaling. The authors further observed that nelfinavir promoted increased tissue oxygenation and demonstrated anti-proliferative properties [120]. Since hypoxia has been linked with reduced radiation-sensitivity in tumor cells, and increased tumor perfusion is deemed as a way to overcome radiation resistance [119], the role of nelfinavir in enhancing tissue oxygenation has garnered significant interest.

Yang et al. showed nelfinavir-driven downregulation of matrix metalloproteinase-2 (MMP-2) in NSCLC cells [47]. It was shown that protease inhibitors have the potential of downregulating matrix metalloproteinase-9 (MMP-9) and MMP-2 during adipocyte differentiation and in glioblastoma cells [121,122]. Matrix metalloproteinases are important modulators of tumor cell invasion and metastasis; thus, nelfinavir could potentially be used to inhibit tumor progression and metastasis. In functional assays, Xia et al. showed that nelfinavir inhibited cell migration and invasion of cervical cancer cells in vitro, which was enhanced in combination with metformin [34]. In medullary thyroid carcinoma cells, nelfinavir reduced the level of HSP90 client proteins E-cadherin, SRC, and connexin-43 which was associated with inhibited adhesive property of the cancer cells, leading to the reduced spheroid formation and induction of anchorage-dependent cell death (anoikis) [38]. Nelfinavir was also shown to inhibit the invasive property of papillary, follicular, and anaplastic thyroid cancer cells with concomitant reduction in the expression of gap-junction protein connexin-43 and reduced mitochondrial membrane potential [26].

Pancreatic stellate cells are important drivers of desmoplastic reaction—a fibrotic and inflammatory patho-histological change—in pancreatic ductal adenocarcinoma, which is believed to raise radiation resistance in pancreatic cancer [123]. Nelfinavir was reported to sensitize pancreatic cancer cells to radiation with or without the presence of human pancreatic stellate cells (hPSC). Nelfinavir reduced the phosphorylation of focal adhesion kinase (FAK) and Akt in hPSCs. Following administration of nelfinavir in vivo, pancreatic tumors, despite being mixed with hPSC, showed improved response to radiotherapy and delayed their growth kinetics [123]. Cancer stem cells possess self-sustaining capacity and are responsible for relapse and dissemination of disease. Darini et al. demonstrated that nelfinavir, along with ritonavir, saquinavir, and lopinavir, was able to kill Oct-4 expressing cancer stem cells potently [124]. Nelfinavir was also able to decrease the expression of CD209 in monocyte cells, a target required for HIV virions to invade T cells, which could be added to the immunomodulatory anti-cancer properties of the drug [125].

### 2.9. Multidrug-Resistant Efflux Pumps

ATP-binding cassette (ABC) transporters are transmembrane proteins responsible for expelling endogenous substrates (amino acids, inorganic anions, hydrophobic metabolites), and exogenous drugs and their toxic metabolites from the cell. Among the 48 members of the ABC-transporter family, p-glycoprotein (P-gp/MDR1/ABCB1) has been extensively studied and shown to be associated with the emergence of resistance to chemotherapy in multiple cancers by decreasing the intracellular concentration of drugs [126]. Nelfinavir has been proposed to be a chemosensitizing agent based on its P-gp modulatory function. Besse et al. reported overexpression of P-gp in carfilzomib-resistant MM cell lines and primary cells, which was associated with the limited proteasome-inhibitory activity of carfilzomib. Nelfinavir and lopinavir reduced P-gp-mediated efflux of carfilzomib in MM cells indirectly via inhibiting the mitochondria permeability transition pore (mPTP) [87]. Previous attempts to develop and combine P-gp inhibitory drugs in the chemotherapy regimen of MM resulted in undesirable pharmacokinetic events. However, nelfinavir demonstrated positive results in combination with proteasome inhibitors (bortezomib, carfilzomib) against MM patients in clinical trials [20,75,76]. Besse et al. suggested that the P-gp inhibitory property of nelfinavir could play a role during the chemosensitization of MM patients to proteasome inhibitors. Furthermore, the level of P-gp in patients could be used as a prognostic marker to stratify MM patients, likely to be benefitted from nelfinavir-proteasome inhibitor combination [87].

The upregulation of P-gp has been associated with the activation of the PI3K–Akt–mTOR survival pathway [127]. Nelfinavir increased the intracellular level of doxorubicin in doxorubicin-resistant breast cancer cells via inhibiting the function and membrane localization P-gp, which was associated with downregulation of the PI3K–Akt pathway and activation of ER stress [60]. Kim et al. observed nelfinavir-mediated chemosensitization of vincristine-resistant oral squamous cell carcinoma cells back to the antimitotic agent vincristine, which was associated with the induction of late apoptosis and inhibition of P-gp [128].

Increased expression of P-gp confers resistance to CML. Liu et al. demonstrated nelfinavir-mediated sensitization of doxorubicin-resistant CML cells back to doxorubicin and other drugs transported by inhibiting P-gp (colchicine, paclitaxel, imatinib). Nelfinavir also increased the intracellular concentration of doxorubicin in doxorubicin-resistant CML cells, which was associated with inhibition of P-gp. Although the mRNA and protein levels of P-gp were unaltered in response to nelfinavir, the reduction in intracellular ATP level and mitochondrial potential was deemed to be associated with the functional inhibition of ATP-dependent P-gp transporters. Co-administration of glucose during nelfinavir and doxorubicin treatment in doxorubicin-resistant CML cells reduced nelfinavir-mediated sensitization to doxorubicin, further confirming the possible role of ATP-depletion in inhibition of P-gp and efflux of doxorubicin. Additionally, molecular docking simulation indicated the possibility of competitive binding of nelfinavir at the ATP binding site of P-gp, inhibiting its function [45]. Paradoxically, nelfinavir has also shown to act as a substrate of P-gp efflux pump, which can increase the activity of P-gp as a compensatory mechanism [129,130], warranting caution while considering the role of nelfinavir as a P-gp inhibitor for drug sensitization. Among other members of ABC transporter proteins, nelfinavir was shown to interact with breast cancer-resistant protein (BCRP/ABCG2) [131] and multidrug-resistant protein 4 (MRP4/ABCC4) [132].

### 2.10. Summary of Mechanisms of Action of Nelfinavir as an Anti-Cancer Agent

**Figure 1:**
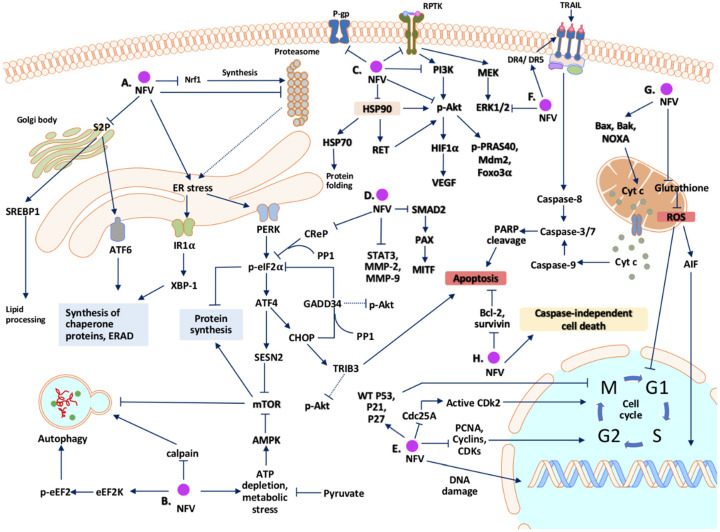
(**A**) Nelfinavir inhibits the Nrf1-dependent synthesis of proteasome subunits and inhibits the proteasome, leading to accumulation of misfolded proteins that activate IRE1α and PERK arms of the UPR. PERK activation leads to modulation of protein synthesis and cell death. Nelfinavir also inhibits S2P in the Golgi apparatus causing accumulation of un-cleaved ATF6 and SREBP. (**B**) Nelfinavir activates autophagy by inhibiting mTOR and by activating eEF2K; however, nelfinavir-mediated inhibition of calpain may impair autophagy as well. (**C**) Nelfinavir can inhibit HSP90 and its interaction with client proteins Akt, RET and HSP70. Nelfinavir can also cause inhibition of P-gp efflux pump and receptor protein tyrosine kinase (RPTK). Inhibition of the PI3K–Akt pathway leads to inhibition of VEGF hindering angiogenesis. (**D**) Nelfinavir inhibits phosphatase CReP, STAT3, MMP-2/9, and the SMAD2 pathway. (**E**) Nelfinavir promotes DNA damage and can lead to cell cycle arrest by modulating components of cell cycle. (**F**) Nelfinavir increases the expression of DR4/5 to enhance TRAIL sensitivity and activates the extrinsic apoptotic pathway. Nelfinavir also inhibits the MAPK pathway. (**G**) Nelfinavir decreases the mitochondrial membrane potential and activates the intrinsic apoptotic cascade. Nelfinavir also inhibits glutathione to increase the production of ROS, leading to cell cycle arrest. Nelfinavir-mediated translocation of the apoptosis-inducing factor (AIF) from the mitochondria to the nucleus contributes to cell death. (**H**) Nelfinavir inhibits the expression of anti-apoptotic proteins facilitating apoptosis and contributing to caspase-independent cell death.

All mechanisms of action previously described for nelfinavir as an anti-cancer agent are summarized below in Figure 1.

## 3. Anti-Tumor Effects of Nelfinavir: Preclinical Evidences In Vivo

The anti-tumor effects of nelfinavir have been tested on different mouse xenograft models in order to assess the translatability of the evidences obtained through cell-based experiments. The data regarding nelfinavir treatment, with or without a co-treatment, on in vivo cancer models are compiled in Table 1.

## 4. Current Status of Clinical Trials

Promising preclinical data regarding nelfinavir, as a single agent or in combination with other cancer therapies, on multiple cancers, prompted a series of clinical trials. For instance, Rengan and colleagues reported the outcome of a phase I/II trial of nelfinavir with concurrent chemoradiotherapy on locally advanced unresectable stage IIIa/IIIb NSCLC [133,134]. In the phase I study, the maximum tolerated dose of nelfinavir was determined to be 1250 mg per oral route twice daily. Nelfinavir was administered 7 to 14 days prior to and concurrently with cisplatin, etoposide, and radiotherapy at a 66.6 Gy dose. No significant predetermined dose-limiting toxicity was observed. Five of the nine evaluable patients showed complete response, whereas the remaining four patients showed partial response in post-treatment positron emission tomography (PET)-derived metabolic evaluation [133]. The phase I study progressed into a phase II study where 35 patients with locally advanced unresectable stage IIIa/IIIb NSCLC were treated with nelfinavir with concurrent chemoradiotherapy. Observed median survival was 41.1 months and a median progression-free survival was 11.7 months without any unexpected grade 3 or 4 toxicities beyond those of standard chemoradiotherapy [134].

Radiotherapy is a front-line management option for inoperable locally advanced pancreatic cancer (LAPC); however, resistance to radiation is frequent and local disease progression leads to the demise of patients. In the preclinical setting, nelfinavir was shown to increase the sensitivity to radiation via the downregulation of Akt [96], reducing hypoxia [103], and improving tumor microvasculature [120]. Brunner et al. first reported a phase I trial with the use of nelfinavir in conjunction with chemoradiotherapy in inoperable LAPC patients [107]. In this study, 12 patients started nelfinavir three days before the initiation of radiation therapy and chemotherapy with cisplatin and gemcitabine. Of the 10 evaluable patients, 5 showed complete metabolic response in PET and 6 underwent secondary resection. The median overall survival was 18 months, and most patients showed downregulation of p-Akt in PBMCs. Nelfinavir did not contribute to additional or unexpected toxicity to the regimen [107]. The study escalated into phase II, where 23 patients with estimated life expectancy ≥ 12 weeks received nelfinavir 1250 mg twice daily prior to and concurrently with radiotherapy and chemotherapy (cisplatin and gemcitabine) [135]. In this study, the median overall survival time was 17.4 months, (90%CI: 12.8–18.8%) and one-year overall survival rate was 73.4% (90% CI: 54.5–85.5%). Four of the six recruited patients for a sub-study showed reduced hypoxia in 18F-fluoromisonidazole positron emission tomography (FMISO-PET) with a concurrent increase in computed tomography (CT) perfusion denoting increased blood flow. Additionally, 8 of 13 evaluable patients demonstrated the downregulation of p-Akt following initial nelfinavir treatment. However, a high incidence of grade 3 or above gastrointestinal toxicity raised concern, which was attributed to the gemcitabine-cisplatin combination with concurrent large-field radiotherapy [135,136]. To address the need to optimize the chemoradiation regime for LAPC, a large-scale multicenter randomized study SCALOP-2 began in March 2016. The study aims at investigating the benefit of induction-chemotherapy by gemcitabine and nab-paclitaxel followed by escalating doses of radiation with or without the radiosensitizer nelfinavir [136]. Recently, Lin et al. reported two trials testing the simultaneous use of nelfinavir with stereotactic body radiotherapy (SBRT) on patients having locally advanced or unresectable pancreatic adenocarcinoma [137,138]. In the phase I study, patients received three-week cycles of gemcitabine/leucovorin/fluorouracil followed by combinations of nelfinavir and escalating doses of radiation therapy. In this study, a median overall survival was estimated to be 14.4 months, and the maximum tolerated dose combination was deemed SBRT (40 Gy)/nelfinavir (1250 BID) [137]. Additionally, in a prematurely terminated trial, Lin et al. tested a chemoimmunotherapy combination gemcitabine/leucovorin/fluorouracil/oregovomab followed by SBRT (40 Gy)/nelfinavir (1250 BID) in LAPC patients [138].

In a few studies, nelfinavir was tried as a monotherapy, unlike the mostly tested regimen of nelfinavir in combination with chemotherapy and with or without radiation therapy. Hoover et al. reported a phase II clinical trial in patients with recurrent adenoid cystic carcinoma who no longer responded to the available standard therapeutic options. Patients received doses of 1250 mg of nelfinavir twice daily; however, the progression-free survival did not improve significantly [109]. Conversely, in a phase I study conducted by Pan et al., 6 patients out of 20 (30%), having recurrent, metastatic or unresectable liposarcoma, showed clinical benefits at different dose levels of nelfinavir [139]. Nelfinavir was reasonably tolerated without any dose-limiting toxicity, and dose escalation was effective up to 3000 mg due to auto-induction of increased plasma clearance at higher doses [139]. Blumenthal et al. investigated the effects of nelfinavir monotherapy on adults having advanced solid refractory tumors of different origins [57]. Patients showed well tolerability to nelfinavir with manageable toxicities and the maximum tolerated dose was determined at 3125 mg. Dose-limiting toxicity was reported as grade 4 neutropenia at a high dose level (3750 mg), which was reversible quickly upon temporary discontinuation of the treatment. Out of 28 patients, one showed partial response, three showed minor response and six showed stable disease on tumor evaluation. Importantly, this study reported the beneficial effect of nelfinavir on a neuroendocrine tumor (NET). Patients showed decreased p-Akt, enhanced p-eIF2α and enhanced expression of ATF3 and CHOP analyzed from PBMCs following nelfinavir treatment [57].

Decreased UPR, especially silencing of IRE1α/XBP1 in MM cells has been shown to confer resistance to proteasome inhibitor bortezomib [140]. In a phase I study, Driessen et al. observed the upregulation of UPR proteins in response to nelfinavir—with or without bortezomib—in PBMCs of advanced MM patients [75]. Among six bortezomib and lenalidomide refractory MM patients, three showed partial response, and two demonstrated minor response to the combination of nelfinavir (2 × 2500 mg) and bortezomib. Nelfinavir also showed mild inhibition of proteasome activity, which was further enhanced by bortezomib [20,75]. In a phase II trial 34 patients of bortezomib-refractory MM, a twice daily dose of 2500 mg of nelfinavir lead to an objective response rate of 65% (90% CI, 49–76%) and was observed with 12 weeks of progression-free survival and a median overall survival of 12 months [20]. Recently, Hitz et al. reported a regime of nelfinavir/lenalidomide/dexamethasone, a triad of orally given drugs, tried on 29 patients with lenalidomide refractory MM [76]. Ten of the 29 patients had lenalidomide-bortezomib double-refractory MM; 16 patients showed minor response or better (55%, 95% CI 36–74%), and 9 patients showed partial response (31%, 95% CI 15–51%), with median overall survival of 21.6 months. Lenalidomide and nelfinavir both act as substrates for multidrug-resistant 1 (MDR-1) pump which may have caused competing interaction and inhibited drug efflux, thereby increasing intracellular concentration and clinical effects [76].

Hill et al. conducted a clinical trial of combining nelfinavir and radiotherapy on 10 patients having advanced metastatic rectal cancer. Unlike previous studies, nelfinavir (1250 mg twice daily) was combined with hypofractionated radiotherapy without the addition of chemotherapy. Five patients demonstrated tumor regression as per MRI imaging, and dynamic imaging (p-CT, DCI-MRI) hinted increased perfusion in the tumor area [141]. In another small cohort of 11 patients, Buijsen et al. investigated the tolerability of nelfinavir with standard radiotherapy and capecitabine (825 mg/2) in locally advanced rectal cancer patients. Three patients showed pathological complete response and 4 other patients showed major response. Diarrhea appeared to be the most frequent adverse event, which was speculated to be related to the high plasma level of nelfinavir due to inhibition of CYP2C9—a metabolizer enzyme of nelfinavir—by capecitabine. The maximum tolerated dose of nelfinavir was deemed 750 mg twice daily [142]. In patients diagnosed with glioblastoma multiforme (GBM), in order to determine the dose limiting toxicity and maximum tolerated dose of nelfinavir, in conjunction with temozolomide and radiotherapy, Alonso-Basnata and colleagues conducted a phase I trial on 21 patients. Nelfinavir was deemed to be safe when administered with temozolomide (75 mg/m^2^) and radiotherapy (6000 cGy to the gross tumor volume), and the maximum tolerated dose was 1250 mg, similar to the standard dose of HIV infected patients [143]. The bulk of clinical trial data are compiled in Table 2.

## 5. Conclusions

Despite promising advancement in cancer therapeutics, the emergence of novel mutations and resistance to chemoradiotherapy results in low survival rates. Additionally, increased cost and requirement of highly efficient setup for chemoradiotherapy hinders patient access to efficacious treatments within low-income populations and areas with limited resources. Drug repurposing for cancer therapy can maximize the optimal use of the existing drug repertoire and lower the time and cost of developing new therapies. An anti-HIV protease inhibitor, nelfinavir, has been proven efficacious, as a monotherapy, against a variety of cancers in both preclinical settings and clinical trials. Furthermore, nelfinavir sensitized cancer cells to existing regimens of chemotherapy and radiotherapy. Nelfinavir has been in use as an anti-infective agent against HIV for more than two decades, demonstrating good safety profile and tolerable toxicities. The main toxicities associated with long-term frequent dosing of nelfinavir are impairment of glucose metabolism and lipodystrophy, which are reversible upon discontinuation; hence, the potential anti-tumor benefits may outweigh the associated risk of toxicities. As nelfinavir is an orally administered drug, it may lead to good patient compliance and be a preferred drug of choice in resource-limited settings.

Nelfinavir can target a number of mechanisms in mammalian cancer cells; however, definitive identification of the primary cellular target responsible for anti-tumor efficacy is still needed. Analysis of reports indicating probable intracellular pathways suggests that the mechanisms to impart anti-cancer properties by nelfinavir may be cell type and cancer-specific. A number of phase I and II clinical trials have proven the safety, tolerability and positive outcome of nelfinavir in cancer patients, with or without co-treatments, especially against pancreatic cancer, NSCLC, and MM [75,107,134]. So far, the completed clinical trials have been single arm and open-labelled involving small cohorts and the available data warrants randomized controlled trials on larger population groups. Accordingly, two large scale randomized trials are currently ongoing to test the efficacy of nelfinavir with radiotherapy against locally advanced pancreatic cancer (NCT02024009) and cervical cancer (NCT03256916).

Anti-infective dosing of nelfinavir in HIV-infected patients results in a maximum plasma concentration of 7–9 μmol/L, and reports have shown that anti-cancer effects can be achieved within this range [16,19,21]. However, higher plasma concentration may be needed to elicit anti-cancer properties by nelfinavir against some cancers [41]. As nelfinavir is an inducer and substrate of metabolic enzyme CYP34A, autoinduction of plasma clearance in high doses is initiated, which prevents increment of plasma concentration during dose escalation, leading to non-linear pharmacokinetics [139]. Enhanced plasma concentration and tissue availability of nelfinavir can be achieved through molecular modification, drug combination, or nano-particle-based administration. Molecular modification through nitric oxide (NO) hybridization of HIV-PIs have emerged as an alternative strategy to increase the anti-cancer efficacy in lower doses, especially in case of saquinavir [4]. Metabolism of nelfinavir by the enzyme CYP2C19 yields the pharmacologically active metabolite M8 responsible for suppressing the viral replication. M8 has also shown comparable anti-tumor activity to nelfinavir [61]. Kattel et al. reported enhanced systemic exposure of nelfinavir due to genetic polymorphism of CYP2C19 in locally advanced pancreatic cancer patients, suggesting that stratification of patients according to the genotype could identify the population likely to be benefitted from nelfinavir treatment [145].

Overall, the anti-tumor effects of nelfinavir have been tested on an array of cancers, with positive results rationalizing its suitability as a potential candidate for drug repurposing for cancer.

## Figures and Tables

**Table 1:** Anti-tumor effects of nelfinavir in animal models.

Publications (et al.)	Cancer Type	Animal Background	Cells and Method of Xenograft	Dosing of Nelfinavir ± Co-Treatment	Time	Main Result(s)
Al Assar, 2016 [123]	Pancreatic cancer	Female nude mice	PSN-1, SC ^1^, Flank	20 mg/kg, IP ^2^ ± RT ^3^ 3.5 Gy	20 d	Overcoming radioprotective effect of pancreatic stellate cells
Bono, 2011 [37]	Multiple myeloma	NOD/SCID ^4^ mice	U266-Luc ^5^, SC, Flank	75 mg/kg, IP	21 d	Reduced tumor burden
Chakravarty, 2016 [60]	Breast cancer	Female athymic nude BALB/c mice	MCF-Dox ^6^, 4th inguinal mammary gland (orthotopic)	20 mg/kg, IP ± Dox 2 mg/kg	6 w	Reduced tumor growth and p-AKT
Cuneo, 2007 [102]	Lung cancer	C7/BL6 mice	Lewis lung carcinoma, SC, hind limb	30 mg/kg, oral ± RT 2 Gy	3–5 d	Reduced vascular density and angiogenesis
Davis, 2016 [42]	Cervical cancer	Female athymic nude mice	ME-180, ME-180 CPR ^7^, SC, alternate flanks	250 mg/kg/d, gastric gavage	21 d	Reduced tumor growth of both cisplatin sensitive and resistant cells
De Gassart, 2016 [67]	Spontaneous	Immuno-compromised AGR 129 mice	eEF2K ^8^ WT ^9^ HRasV12, eEF2k^−/−^ HRasV12, SC. Alternate flanks	100 mg/kg, IP		Tumor growth inhibition in response to nelfinavir in eEF2K WT mice but not in eEF2K-deficient mice
Escalante, 2013 [81]	Multiple myeloma	SCID mice	MM.1S, SC	50 mg/kg, oral gavage ± BZ ^10^ 1 mg/kg, IV tail vein	Until 10% wt ^11^ loss	Complete tumor regression in combination group
Gills, 2007 [19]	Lung cancer	Balb/cAnCr *nu/nu* mice, athymic nude mice	H157, A548; SC, shoulder and rear flanks	50–100 mg/kg, IP; or 100 mg/kg gastric gavage	10–20 d	Tumor growth delay, ER stress, autophagy
Guan, 2011 [61]	Liposarcoma	SCID mice	Lisa-2, SC, heterotopic model	500 mg/kg/d, diet	41 d	Reduced tumor growth
Gupta, 2007 [50]	Meningioma	Male athymic *nu/nu* mice	IOMM-Lee, SC, right flank	150 mg/kg/d, oral ± Imatinib 100 mg/kg/d	23 d	Combined treatment caused tumor growth reduction, ER stress, apoptosis and reduced level of survivin
Gupta, 2005 [96]	Head and neck cancer, bladder cancer	NCr-*nu/nu* mice	SQ20B (EGFR mutated), T24 (HRas mutated), SC, hind flank	0.6 mg/day, continuous release pellets ± RT 6–8 Gy	Time to reach 1000 mm^3^	Combined treatment caused tumor regrowth delay
Jiang, 2007 [100]	Glioblastoma	Female NCr-*nu/nu* mice	U87MG (PTEN deficient), SC, flank	79 mg/kg/day, diet ± RT 6 Gy	Time to reach 1000 mm^3^	Combined treatment caused tumor growth delay; nelfinavir reduced p-Akt
Johnson, 2018 [70]	Tuberous sclerosis complex	NOD/SCID female mice	ELT3-V3 (*Tsc2^−/−^)*, SC, right flank	30–50 mg/kg, IP ± BZ 0.3–0.5 mg/kg	17 d	Combined treatment caused tumor growth reduction, ER stress, apoptosis
Kawabata, 2012 [56]	NSCLC ^12^, multiple myeloma	Athymic NCr *nu/nu* mice	H157, RPMI8226, SC, both rear flanks	50 mg/kg, IP ± BZ 0.5 mg/kg	11–17 d	Combined treatment caused tumor growth reduction, ER stress, apoptosis
Kimple, 2010 [101]	Pancreatic cancer	Athymic BALB/c nude mice	Capan-2, SC, flanks	150 mg/kg, Oral gavage ± RT 200 cGy/day	10 d	Combined treatment caused tumor growth reduction; nelfinavir reduced p-Akt
Mathur, 2014 [46]	Castration-resistant prostate cancer	Athymic nude mice	C4–2B, SC	DTX ^13^ (10 mg/kg), ± [nelfinavir (20 mg/kg) and curcumin (100 mg/kg)]	4 w	Triple combination caused tumor growth delay and apoptosis
Okubo, 2018 [29]	Renal cancer	BALB/c male nude mice	Caki-2, SC	25 mg/kg, IP ± PAN ^14^ (2 mg/kg)	11 d	Combined treatment caused tumor growth reduction, ER stress, apoptosis and histone acetylation
Pore, 2006 [103]	Lung cancer, head neck squamous cell cancer	BALB/c NCr *nu/nu* mice	A549, SQ20B, SC, flank	79 mg/kg/d, diet; ± RT 8 Gy	Time to reach 1000 mm^3^	Combined treatment reduced tumor growth; nelfinavir reduced angiogenesis and VEGF ^15^
Pore, 2006 [104]	Glioblastoma	BALB/c NCr *nu/nu* mice	U87, SC	40 mg/kg/d; diet	5 d	Reduced angiogenesis
Pyrko, 2007 [58]	Glioblastoma	Male athymic *nu/nu* mice	U87, SC	40 mg/kg/d (short-term), 120 mg/kg/d (long-term); gastric gavage	96 h (short- term), 6 w (long-term)	Tumor growth reduction, ER stress, apoptosis
Qayum, 2009 [120]	Fibrosarcoma, Laryngeal cancer	SCID mice	HT1080, SQ20B, SC, hind leg	20 mg/kg, IP	2 w	Reduced tumor hypoxia, increased tumor blood flow, normalized tumor vascular morphology
Shim, 2012 [88]	Breast cancer	BABL/c NCr *nu/nu* mice	HER2 ^16^ positive: HCC1954, BT474; HER2 negative: HCC1937, MDA-MB-231, SC	25 mg/kg, IP; 40 mg/kg, oral	30 d	Nelfinavir selectively inhibited the growth of HER2-positive tumors and decreased expression of HER2
Smith, 2016 [113]	Melanoma	Nude mice	A375, M249-R4, SC	25 mg/kg/qd, oral gavage ± MEKi ^17^ (25 mg/mg/qd) or BRAFi ^18^ (25 mg/kg/qd)	21–33 d	Combined treatment caused reduction in tumor growth and expression of PAX and MITF ^19^
Thomas, 2012 [41]	Breast cancer	Athymic mice	MDA-MB-468 (TNBC ^20^), MCF-7, SC, flank	5 mg/kg/d, gavage ± Celecoxib (2 mg/kg/d) ± CQ ^21^ (10 mg/kg/d)	3–5 d	Triple combination caused tumor growth reduction, ER stress and apoptosis
Vandewynckel, 2016 [49]	Hepatocellular carcinoma	WT 129s2/SvPasCrl mice injected with DEN ^19^ (orthotopic model); Athymic nude mice: Foxn1nu/foxn1nu (Xenograft model)	HepG2, SC, right flank	OZ ^22^ (30 mg/kg/d), intragastric ± nelfinavir (250 mg/kg/d), IP or salubrinal (1 mg/kg/d), IP	4 w	Decreased tumor growth and increased apoptosis in both orthotopic and xenograft models
Xia, 2017 [34]	Cervical cancer	Female BALB/c nude mice	SiHa, SC, left flank	0.4 mg/kg/d, IP ± metformin 100 mg/kg/d	24 d	Reduced tumor growth and PI3K ^23^ expression and increased expression of p53 and p21 in response to either monotherapy or combined therapy
Xia, 2019 [117]	Cervical cancer	Female BABLB/c nude mice	SiHa, SC, left flank	0.4 mg/kg/d, IP ± metformin 100 mg/kg/d	25 d	Combined treatment caused tumor growth reduction and enhanced level of sirtuin-3 and MICA ^24^, suggesting NK ^25^ cell-mediated lysis
Xiang, 2015 [33]	Cervical cancer	BALB/c nude mice	HeLa, SC, back	1 mg/mouse, IP	20 d	Tumor growth reduction, increased apoptosis, nuclear localization of AIF ^26^
Yang, 2006 [47]	NSCLC	BALB/c triple-deficient male nude mice	NCI-H460, SC, bilateral	60 mg/kg, oral gavage	3 w	Tumor growth reduction, apoptosis
Yang, 2005 [48]	Prostate cancer	Immunodeficient BALB/c nude mice	LNCaP, SC, bilateral	60 mg/kg, oral gavage	3 w	Tumor growth reduction, reduced serum level of PSA ^27^, increased fibrosis and inflammatory cells
Zeng, 2011 [105]	Pituitary adenoma	Female nude mice	GH3, SC, right flank	5 μM, oral gavage ± RT 6 Gy	Until tumor size 4×	Tumor growth reduction, reduced phospho-S6

^1^ Subcutaneous. ^2^ Intraperitoneal. ^3^ Radiotherapy. ^4^ Non-obese diabetic/severe combined immunodeficiency. ^5^ Luciferase. ^6^ Doxorubicin. ^7^ Cisplatin resistant. ^8^ Eukaryotic elongation factor 2 kinase. ^9^ Wild type. ^10^ Bortezomib. ^11^ Weight. ^12^ Non-small-cell lung carcinoma. ^13^ Docetaxel. ^14^ Panobinostat. ^15^ Vascular endothelial growth factor. ^16^ Human epidermal growth factor receptor 2. ^17^ Mitogen-activated protein kinase kinase inhibitor. ^18^ BRAF inhibitor. ^19^ Microphthalmia-associated transcription factor; melanoma transcription factor. ^20^ Triple-negative breast cancer. ^21^ Chloroquine. ^22^ Oprozomib. ^23^ Phosphoinositide-3 kinase. ^24^ Major histocompatibility complex class I chain-related gene A. ^25^ Natural Killer. ^26^ Apoptosis-inducing factor. ^27^ Prostate-specific antigen.

**Table 2:** Updated clinical trial list including nelfinavir (2020).

NCT Number	Phase	Cancer Type	Concurrent Therapy	Timeline	Status	Total Patients	Objective	Ref
NCT01485731	I	Cervical cancer	Cisplatin, RT ^1^	January 2012–February 2015	C ^2^	8	Estimate of adverse event, MTD ^3^	
NCT00589056	I/II	Stage III NSCLC ^4^	Cisplatin, etoposide, RT	June 2007–March 2012	C	55	DLT ^5^, MTD	[134]
NCT01079286	I	Renal cancer	Temsirolimus	June 2008–May 2011	C	18	PK ^6^, PD ^7^, dose escalation	
NCT02363829	I	LA ^8^ Cervical Cancer (Stage II–VA)	Cisplatin, Pelvic RT	February 2015–February 2020	C	6	Number of AE ^9^	
NCT01086332	I/II	Locally advanced pancreatic cancer (LAPC)	Gemcitabine, RT	May 2009–July 2015	T ^10^	7	DLT	
NCT00704600		Colorectal cancer	Capecitabine, Preoperative RT	September 2008–July 2013	C	15	DLT, MTD	[142]
NCT01447589	I/II	NSCLC	Radical radiotherapy	February 2012–October 2012	W ^11^	-	MTD, AE	
NCT01445106	I	Solid tumors	_	December 2006–May 2011	C	28	MTD, DLT, PK, PD, anti-tumor response, blood markers	[57]
NCT01065844	II	Adenoid cystic head and neck carcinoma	_	October 2009–November 2017	C	15	Tumor progression	[109]
NCT01068327	I	Pancreatic cancer (adeno-carcinoma/Stage III)	Gemcitabine hydrochloride, leucovorin calcium, fluorouracil, RT	November 2007–February 2015	C	46	DLT, MTD, evaluate surgical resection rate, pathological and radiological response	[137]
NCT04169763	I	Vulvar cancer (Stage II–IVA)	Cisplatin, external beam radiation	March 2020–December 2023	NR ^12^	18 est. ^13^	DLT, safety, dose for phase II	
NCT01108666	II	Inoperable NSCLC (Stage III)	Cisplatin, paclitaxel, etoposide, proton beam radiation	March 2010–December 2018	T	8	MTD, toxicity, feasibility of proton beam, clinical efficacy	
NCT02024009	I/II	Non-metastatic LAPC	RT, nab-paclitaxel, gemcitabine, capecitabine,	March 2016–August 2020	R ^14^	289 est.	OS ^15^, PFS ^16^, toxicity, QL ^17^	[136]
NCT03422874	I	Lymphoma	Ixazomib (MLN9708)	August 2016–August 2017	W	_	MTD, toxicity, PK, PD	
NCT01959672	II	LAPC	Gemcitabine hydrochloride, leucovorin calcium, fluorouracil, oregovomab, RT	September 2013–December 2018	C	11	Evaluate efficacy and safety of neoadjuvant chemotherapy followed by RT+ nelfinavir	[138]
NCT01164709	I	Advanced hematologic malignancies	Bortezomib	July 2010–November 2013	C	18	DLT, objective response, AE	[75]
NCT03050060	II	Advanced melanoma, lung and kidney cancer	Atezolizumab, nivolumab, pembrolizumab, RT	June 2017–December 2021	S ^18^	120 est	RR ^19^, OS, PFS, AE, immune correlative studies	
NCT02080416	I	Gamma-herpes related tumor	_	July 2014–February 2016	T	1	Lytic activation of viral gene expression by nelfinavir	
NCT01925378	II	Cervical dysplasia	_	July 2012–December 2022	R	10 est.	Efficacy of nelfinavir	
NCT00791336	II	NSCLC	RT, cisplatin, etoposide	August 2008–March 2011	T	1	Pathologic complete response	
NCT00915694	I	GBM ^20^	Temozolomide, RT	April 2009–December 2015	T	15	MTD, DLT, PFS, OS	[144]
NCT03256916	III	Carcinoma cervix (Stage III)	Cisplatin, pelvic RT	January 2018–September 2025	R	300	Improvement in 3 year disease-free survival	
NCT03829020	I	Relapsed or refractory multiple myeloma	Bortezomib, metformin	April 2019–August 2021	R	36 est.	MTD, AE, hematological response	
NCT02188537	II	Proteasome inhibitor-refractory myeloma	Bortezomib, dexamethasone	December 2014–April 2018	C	34	RR, AE, QL	[20]
NCT01555281	I/II	Multiple myeloma	Lenalidomide, dexamethasone	February 2012–December 2021	AnR ^21^	33	DLT, ORR ^22^, OS, PFS	[76]
NCT00233948	I/II	Liposarcoma	_	March 2006–July 2013	T	29	DLT, MTD, ORR	
NCT00002185	II	Kaposi sarcoma	_	_	C	20	Safety and efficacy	
NCT02207439	II	Head and neck carcinoma	RT, platinum-based chemotherapy	July 2014–December 2020	AnR	28	Determine locoregional control	
NCT03077451	II	Kaposi sarcoma	_	March 2017–October 2020	AnR	36	Efficacy of dose escalation	
NCT00694837	I	GBM	Temozolomide, RT	March 2009–January 2013	C	6	MTD, toxicity	
NCT01020292	I	Glioma	Temozolomide, RT	April 2009–December 2017	C	31	MTD, DLT, PFS, OS	
NCT00003008	II	Sarcoma	Indinavir, saquinavir, ritonavir, paclitaxel	June 1997–June 2006	C	33	Role of HIV-PIs in plasma clearance of paclitaxel	

^1^ Radiotherapy. ^2^ Completed. ^3^ Maximum tolerated dose. ^4^ Non-small-cell lung carcinoma. ^5^ Dose limiting toxicity. ^6^ Pharmacokinetics. ^7^ Pharmacodynamics. ^8^ Locally advanced. ^9^ Adverse events. ^10^ Terminated. ^11^ Withdrawn. ^12^ Not recruiting. ^13^ Estimated. ^14^ Recruiting. ^15^ Overall survival. ^16^ Progression-free survival. ^17^ Quality of life. ^18^ Suspended. ^19^ Response rate. ^20^ Glioblastoma multiforme. ^21^ Active, not recruiting. ^22^ Overall response rate.

## Abbreviations

ABC	ATP-binding cassette
AIDS	Acquired immunodeficiency syndrome
AIF	Apoptosis-inducing factor
AML	Acute myeloid leukemia
AMPK	5′-AMP-activated protein kinase
ATF3	Activating transcription factor 3
ATF6	Activating transcription factor 6
ATP	Adenosine triphosphate
BrdU	Bromodeoxyuridine
CDK	Cyclin-dependent kinase
CHOP	CCAAT enhancer-binding protein homologous protein
CML	Chronic myeloid leukemia
CT	Computer tomography
DR	Death receptors
DEN	Diethylnitrosamine
DMC	Dimethylcelecoxib
eIF2α	Eukaryotic initiation factor 2α
eEF2	Eukaryotic elongation factor
eEF2K	Eukariotic elongation factor 2 kinase
EGFR	Epidermal growth factor receptor
ER	Endoplasmic reticulum
ERp44	Endoplasmic reticulum resident protein 44
ERO1-Lα	Endoplasmic reticulum oxidoreductin-1-like protein α
FACS	Fluorescence-activated cell sorting
FADD	Fas-associated protein with death domain
FAK	Focal adhesion kinase
FAS	Fatty acid synthase
FDA	Food and Drug Administration
FMISO-PET	18F-fluoromisonidazole positron emission tomography
GADD34	Growth arrest and DNA damage inducible protein 34
GBM	Glioblastoma multiforme
GFP	Green fluorescent protein
GRP78	Glucose-regulated protein of 78 kDa
GSH	Glutathione
HAART	Highly active antiretroviral treatment
HCC	Hepatocarcinoma cells
HDAC	Histone deacetylase
HER2	Human epidermal factor receptor 2
HIF1α	Hypoxia-inducible factor 1α
HIV	Human immunodeficiency virus
HPV	Human papillomavirus
hPSC	Human pancreatic stellate cells
HSP90	Heat shock protein 90
H_2_O_2_	Hydrogen peroxide
IGFR	Insulin-like growth factor receptor
IRE1α	Inositol-requiring enzyme 1-α
ISR	Integrated stress response
KS	Kaposi’s sarcoma
LAPC	Locally advanced pancreatic cancer
MAPK	Mitogen-activated protein kinase pathway
3MA	3-methyladenosine
mTOR	Mammalian target of rapamycin
3-MA	3-methyladenine
MDR	Multidrug resistance
MDR1	Multidrug-resistant 1
MRP-4	Multidrug resistance protein 4
MEF	Mouse embryonic fibroblasts
MM	Multiple myeloma
mPTP	Mitochondria permeability transition pore
MMP-2	Matrix metalloproteinase-2
MMP-9	Matrix metalloproteinase-3
NAC	N-acetylcysteine
NET	Neuroendocrine tumor
NO	Nitric oxide
NSCLC	Non-small-cell lung carcinoma
PARP	Poly ADP-ribose polymerase
PBMC	Peripheral blood mononuclear cells
PCNA	Proliferating cell nuclear antigen
PDI	Protein disulfide isomerase
PE	Phosphatidyl ethanolamine
PERK	Protein kinase RNA-like endoplasmic reticulum kinase
PET	Positron emission tomography
P-gp	P-glycoprotein
PI	Protease inhibitors
PTEN	Phosphate and tensin homologue
RIP	Regulated intramembrane proteolysis
RNA	Ribonucleic acid
ROS	Reactive oxygen species
SBRT	Stereotactic body radiotherapy
SIRT3	Sirtuin-3
STAT3	Signal transducer and activator of transcription 3
S1P	Site-1 protease
S2P	Site-2 protease
SESN2	Sestrin-2
SRC	Tyrosine kinase *Src*
SREBP1	Sterol regulatory binding protein-1
siRNA	Small interference RNA
TNBC	Triple-negative breast cancer
TNF	Tumor necrosis factor
TRAIL	Tumor necrosis factor-related apoptosis-inducing ligand
TRIB-3	Tribbles homolog-3
TSC	Tuberous sclerosis complex
TUNEL	Terminal deoxynucleotidyl transferase (TdT) dUTP Nick-End Labeling
UPR	Unfolded protein response
VEGF	Vascular endothelial growth factor
XBP-1	X-box binding protein-1
